# Clear Improvement in Real-World Chronic Myeloid Leukemia Survival: A Comparison With Randomized Controlled Trials

**DOI:** 10.3389/fonc.2022.892684

**Published:** 2022-07-14

**Authors:** Claudia Vener, Silvia Rossi, Pamela Minicozzi, Rafael Marcos-Gragera, Hélène A. Poirel, Marc Maynadié, Xavier Troussard, Gabriella Pravettoni, Roberta De Angelis, Milena Sant, the EUROCARE-6 Working Group, M. Hackl

**Affiliations:** ^1^ Analytical Epidemiology and Health Impact Unit, Department of Research, Fondazione Istituto di Ricovero e Cura a Carattere Scientifico (IRCCS) Istituto Nazionale dei Tumori, Milan, Italy; ^2^ Department of Oncology and Hemato-Oncology, University of Milan, Milan, Italy; ^3^ Department of Oncology and Molecular Medicine, Istituto Superiore di Sanità, Rome, Italy; ^4^ Cancer Survival Group, Department of Non-Communicable Disease Epidemiology, London School of Hygiene and Tropical Medicine, London, United Kingdom; ^5^ Epidemiology Unit and Girona Cancer Registry, Oncology Coordination Plan, Department of Health, Autonomous Government of Catalonia, Catalan Institute of Oncology, Girona Biomedical Research Institute (IdiBGi), Universitat de Girona, Girona, Spain; ^6^ Biomedical Network Research Centers of Epidemiology and Public Health (CIBERESP), Madrid, Spain; ^7^ Group of Descriptive and Analytical Epidemiology of Cancer, Josep Carreras Leukemia Research Institute, Girona, Spain; ^8^ Belgian Cancer Registry, Brussels, Belgium; ^9^ Côte d'Or Hematological Malignancies Registry, Dijon, France; ^10^ Basse Normandie Cancer Registry, Centre Hospitalier Universitaire (CHU) de Caen, Normandie, Caen, France

**Keywords:** cancer registries, chronic myeloid leukemia (CML), randomized controlled trials (RCTs), real-world data, survival, Europe, tyrosine kinase inhibitor (TKI), population-based studies

## Abstract

Tyrosine kinase inhibitors (TKIs) have been improving the prognosis of patients with chronic myeloid leukemia (CML), but there are still large differences in survival among European countries. This raises questions on the added value of results from population-based studies, which use real-world data, compared to results of randomized controlled trials (RCTs) involving patients with CML. There are also questions about the extent of the findings on RCTs effectiveness for patients in the general population. We compare survival data extracted from our previous systematic review and meta-analysis of CML RCTs with the latest updated population-based survival data of EUROCARE-6, the widest collaborative study on cancer survival in Europe. The EUROCARE-6 CML survival estimated in patients (15–64 years) diagnosed in 2000–2006 vs. 2007–2013 revealed that the prognostic improvement highlighted by RCTs was confirmed in real-world settings, too. The study shows, evaluating for the first time all European regions, that the optimal outcome figures obtained in controlled settings for CML are also achievable (and indeed achieved) in real-world settings with prompt introduction of TKIs in daily clinical practice. However, some differences still persist, particularly in Eastern European countries, where overall survival values are lower than elsewhere, probably due to a delayed introduction of TKIs. Our results suggest an insufficient adoption of adequate protocols in daily clinical practice in those countries where CML survival values remain lower in real life than the values obtained in RCTs. New high-resolution population-based studies may help to identify failures in the clinical pathways followed there.

## Highlights

1. The EUROCARE-6 CML survival estimates revealed that the prognostic improvement highlighted by RCTs was confirmed in the European real-world setting.

2. There are still large differences in CML survival throughout Europe: the prompt introduction of TKIs in daily clinical practice is undelayable.

## 1 Introduction

The European incidence of chronic myeloid leukemia (CML) was about 1.1/100,000 inhabitants (1), increasing to about 4.0/100,000 in patients aged 75–99 at the time of diagnosis. The disease is characterized by the presence of the *BCR-ABL1* fusion gene located in the Philadelphia (Ph) chromosome and is classified as being in a chronic (CP), accelerated (AP), or blastic phase (BP), with the last two phases accounting for about 4% and 3% of cases, respectively (2, 3) and being associated with a worse prognosis (4).

For many years, CML was associated with a poor life expectancy (5), but the 2001 introduction of imatinib mesylate, the first tyrosine kinase inhibitor (TKI) and, more recently, of second- and third-generation TKIs (dasatinib, nilotinib, bosutinib, and ponatinib) has profoundly changed the CML curative-intent treatment, previously based on hematopoietic stem cell transplantation. TKIs have greatly improved CML survival rates and now make it possible to consider CML a chronic disease (6–11). Imatinib was approved as first-line treatment for all CML phases and is now available as a generic drug, as its patent has expired. Dasatinib and nilotinib were approved in 2006–2007 as second-line treatments for patients resistant to, or intolerant of, previous treatments (including imatinib): dasatinib in all CML phases and nilotinib only in the CP or AP. Since 2010–2011, both have been authorized for the first-line treatment of newly diagnosed Ph-positive adult cases of CP CML. Bosutinib was licensed in the United States in 2012 (and in Europe in 2013) for the treatment of adults with CP, AP, or BP CML who are resistant to, or intolerant of, previous treatments with one or more TKIs. In December 2017, the recommendation was extended in the United States to include newly diagnosed adult patients with CP CML. Ponatinib was approved in the United States in 2012 (and in Europe in 2013) for the treatment of adults with CP, AP, or BP CML who are resistant to, or intolerant to, other TKIs and also for the treatment of those with CP, AP, or BP CML who have the T315I mutation, which is known to be involved in resistance to all previous TKIs.

The 5-year survival estimates for patients with CML increased from 1997 to 2008 throughout Europe (particularly after 2000), although with large differences among European countries (10, 12): they increased slightly in Southern Europe, more in the United Kingdom, and considerably more in Northern, Central, and Eastern Europe, although in the latter region, survival remained lower than elsewhere (10). These improvements were plausibly linked to the widespread introduction of targeted and other new treatments (10). There was only a small increase in survival estimates among the elderly, possibly because of the under-use of imatinib (90% of patients aged 20–59 received imatinib, 75% of those aged 60–79, and 46% of those aged ≥80) and the newer TKIs (13). Furthermore, the cancer registry (CR) of Girona showed that the 5-year survival rate in patients with CML treated with TKIs in 1994–2008 was about 80%, compared with 44% among those who were not (14).

Population-based studies including all cases occurring in the region covered by a CR reflect the effectiveness of healthcare services in controlling the disease and are more likely to highlight socioeconomic disparities potentially associated with cancer survival. People who live in more affluent areas have better access to optimal care than those living in deprived areas, and this leads to discrepancies in overall survival (OS) figures (15). Moreover, access to optimal treatment is related to per capita income and healthcare investments (16).

Clinical practice, particularly in oncological settings, often relies on randomized controlled trials (RCTs) because they provide more detailed information than population-based studies. However, the amount of data may be overwhelming (17), and it can be difficult to determine the health systems’ sustainability, in terms of finance and uptake of new practices. As a consequence, oncological organizations have developed frameworks to help clinicians and policymakers quantify the real value of new therapies (17–21). Generalizing trial results to everyday clinical practice is not straightforward because of low overall trial accrual (<5% of all newly diagnosed patients with cancer) and under-representation by age, gender, disease stage, co-morbidities, and socioeconomic status. However, despite these limitations, approved treatments are frequently offered to patients who would have been ineligible for the related trials, but they rarely show the benefits detected in RCTs; furthermore, a survival advantage detected by RCTs is not always subsequently confirmed in real-life setting.

This raises questions as to how the results of population-based studies using real-world data can add to the results of RCTs involving patients with CML and to the findings on the extent of RCTs’ effectiveness for the patient population as a whole. In an attempt to answer these questions, we compared the survival of patients with CP CML participating in RCTs with the data from EUROCARE, the widest collaborative population-based study on cancer survival in Europe (22).

## 2 Materials and Methods

### 2.1 Study Design

We extracted the survival data from the RCTs included in our previous systematic review and meta-analysis comparing first-line imatinib and second- and third-generation TKIs in adults with newly diagnosed CP CML [International Prospective Register of Systematic Review (PROSPERO) Registration No. CRD42016032903] (**Table 1**) (58, 59).

**Table 1:** Summary of the findings of the RCTs included in the meta-analysis.

RCT	No. of patients	Median age (range), years	Males (No., %)	FU (months)	Authors, year	Journal	OS (%) (I/C)						
							12 months	18 months	24 months	36 months	48 months	60 months	72 months
**DASISION** ^*^ **(D) (NCT00481247)**	519	I: 49 (18–78) D: 46 (18–84)	I: 163 (63) D: 144 (56)	12	Kantarjan H.M. et al., 2010 (23)	N Engl J Med^‡^	99.0/97.0	–	–	–	–	–	–
				18	Shah N. et al., 2010 (24)	Blood^§^	-	97.9/96.0	–	–	–	–	–
				24	Kantarjan H.M. et al., 2011 (25)	J Clin Oncol^§^	-	98.0/96.0	–	–	–	–	–
				24	Hochhaus A. et al., 2011 (26)	Blood^§^	-	–	–	–	–	–	–
				24	Hochhaus A. et al., 2012 (27)	J Clin Oncol^§^	-	–	–	–	–	–	–
				24	Kantarjian H.M. et al., 2012 (28)	Blood^‡^	-	–	95.2/95.3	–	–	–	–
				36	Jabbour E. et al., 2014 (29)	Blood^‡^	-	–	–	93.2/93.7	–	–	–
				48	Cortes J.E. et al., 2013 (30)	Blood^§^	-	–	–	–	92.0/93.0	–	–
				60	Cortes J.E. et al., 2014 (31)	Blood^§^	-	–	–	–	–	90.0/91.0	–
				60	Cortes J.E. et al., 2016 (32)	J Clin Oncol^‡^	-	–	–	–	–	90.0/91.0	–
**NCT00070499** ^†^ **(D)**	253	I: 50 (19–89) D: 47 (18–90)	I: 72 (59) D: 74 (60)	12^◦^	Radich J.P. et al., 2012 (33)	Blood^‡^	-	–	–	97.0/97.0	–	–	–
**NordCML006** ^*^ **(D) (NCT00852566)**	46	I: 60 (38–77) D: 54 (29–71)	I: 15 (63) D: 7 (32)	18	Mustjoki S. et al., 2013 (34)	Leukemia^‡^	-	–	–	–	–	–	–
				24	Hjorth-Hansen H. et al., 2013 (35)	Blood^§^	-	–	–	–	–	–	–
				36	Hjorth-Hansen H. et al., 2015 (36)	Eur J Haematol^‡^	-	–	–	–	–	–	–
**ENESTnd** ^*^ **(N) (NCT00471497)**	846	I: 46 (18–80) N300: 47 (18–85) N400: 47 (18–81)	I: 158 (56) N300: 158 (56) N400: 175 (62)	12	Larson R.A. et al., 2010 (37)	J Clin Oncol^§^	-	–	–	–	–	–	–
				12	Saglio G. et al, 2010 (38)	N Engl J Med^‡^	-	–	–	–	–	–	–
				18	Hughes T.P. et al., 2010 (39)	Blood^§^	-	96.9/ 98.5 (N300) 99.3 (N400)	–	–	–	–	–
				24	Kantarjian H.M. et al., 2011 (40)	Lancet Oncol^‡^	-	–	96.3/ 97.4 (N300) 97.8 (N400)	–	–	–	–
				36	Kantarjian H.M. et al., 2012 (41)	Blood^§^	-	–	–	94.0/ 95.1 (N300) 97.0 (N400)	–	–	–
				36	Larson R.A. et al., 2012 (42)	Leukemia^‡^	-	–	–	94.0/ 95.1 (N300) 97.0 (N400)	–	–	–
				36	Hochhaus A. et al., 2013 (43)	Blood^‡^	-	–	–	–	–	–	–
				48	Hughes T.P. et al., 2014 (44)	Blood^‡^	-	–	–	–	93.3/ 94.3 (N300) 96.7 (N400)	–	–
				60	Hochhaus A., 2016 (45)	Leukemia^‡^	-	–	–	–	–	91.7/ 93.7 (N300) 96.2 (N400)	–
				72	Hochhaus A. et al., 2015 (46)	Blood^§^	-	–	–	–	–	–	–
				72	Hughes T.P. et al., 2015 (47)	Haematologica^§^	-	–	–	–	–	–	91.4/ 91.6 (N300) 95.8 (N400)
**BELA** ^†^ **(B) (NCT00574873)**	502	I: 47 (18–89) B: 48 (19–91)	I: 135 (54) B: 149 (60)	12	Cortes J.E., 2012 (48)	J Clin Oncol^‡^	97.0/99.0	–	–	–	–	–	–
				18	Gambacorti-Passerini C., 2011 (49)	J Clin Oncol^§^	-	–	–	–	–	–	–
				24	Brummendorf T.H, 2015 (50)	Br J Haematol^‡^	-	–	95.0/97.0	–	–	–	–
				30	Brummendorf T.H., 2012 (51)	Haematologica^§^	-	–	95.0/97.0	–	–	–	–
				30	Gambacorti-Passerini C., 2014 (52)	Am J Hematol^‡^	-	–	–	–	–	–	–
				48	Cortes J.E., 2016 (53)	Am J Hematol^‡^	-	–	–	–	–	–	–
BFORE* **(B) (NCT02130557)**	536	I: 53 (19–84) B: 52 (18–84)	I: 135 (56) B: 142 (58)	12	Cortes J.E., 2018 (54)	J Clin Oncol^‡^	97.9/99.6	–	–	–	–	–	–
				18	Gambacorti-Passerini C., 2017 (55)	Blood^§^	-	96.6/99.6	–	–	–	–	–
				60	[Brummendorf T.H, 2020^^^ (56)]	Blood^§^						94.6/94.5	
**EPIC** ^†^ **(P) (NCT01650805)**	307	I: 52 (18–86) P: 55 (18–89)	I: 92 (61) P: 97 (63)	12	Lipton J.H, 2016 (57)	Lancet Oncol^‡^	-	–	–	–	–	–	–

RCT, randomized controlled trial; OS, overall survival; FU, follow-up; (−), not evaluated; I/C, imatinib/comparator (B, bosutinib; D, dasatinib; N300, nilotinib of 300 mg; N400, nilotinib of 400 mg; P, ponatinib).

^*^RCT.

^†^Quasi-RCT.

^‡^Full paper.

^§^Abstract.

^◦^36-month OS.

^^^Updated in 2022.

Population-based survival data were extracted from the EUROCARE-6 dataset (22). ICD-O-3 (International Classification of Disease for Oncology, 3rd edition) (60) morphology codes 9863 (CML with no cytogenetic information or CML not otherwise specified, NOS) and 9875 (CML, BCR-ABL1-positive; Ph+ CML) according to HAEMACARE (61) groupings were selected. Code 9876 (Atypical CML, BCR-ABL1-negative; Ph− aCML) was not included.

Quality and completeness of CRs data were evaluated by applying standardized check procedures in conjunction with the ENCR-JRC technical report, to ensure data comparability (62). At the end of the quality checks of the 101 population-based CRs in the EUROCARE-6 database (that provided continuous incidence data for hematological malignancies from January 1, 2000, to December 31, 2013, with follow-up data up until December 31, 2014), only 84 with adequate information for the purposes of the study (sufficient time coverage, follow-up completeness, and morphology accuracy) were selected (**Supplementary Table 1**).

The survival analyses were therefore based on 18,083 eligible CML cases, aged between 15 and 64 (the age selection corresponding to the age of patients with CML usually enrolled in RCTs), provided by 84 regional or national CRs in 28 European countries (**Table 2**). In particular, 8,793 CML cases were diagnosed in 2000–2006 and 9,290 CML cases in 2007–2013. We have defined the threshold of 2006–2007 because it corresponds to the introduction of second-generation TKIs (dasatinib and nilotinib) in clinical practice (first approval in 2006–2007).

**Table 2:** Myeloid malignancies diagnosed in European patients (15–64 years) in 2000–2013 and quality indicators by Cancer Registry (CR). EUROCARE-6 study dataset.

Area/Country		Cancer registry (CR)	Overall period of diagnosis^1^	Myeloid malignancies^2^ 2000–2013					
							CML cases included in survival analysis^3^		
				Cases 2000–2013	% Microscopically Verified (MV)	% Not otherwise specified (NOS)^4^	CML total cases	CML NOS (9863) cases (%)	CML Ph+ (9875) cases (%)
**Northern Europe**	DENMARK	**Denmark**	1978–2014	3,404	98.9	1.3	470	122 (26)	348 (74)
	FINLAND	**Finland**	1978–2013	2,309	90.9	6.9	304	300 (99)	4 (1)
	ICELAND	**Iceland**	1978–2014	78	98.7	1.3	23	22 (96)	1 (4)
	NORWAY	**Norway**	1978–2016	2,557	98.9	1.8	312	283 (91)	29 (9)
**UK and Ireland**	IRELAND	**Ireland**	1994–2012	1,986	98.6	5.7	240	234 (98)	6 (3)
	UK-ENGLAND	**UK-England**	1995–2013	15,100	91.1	5.1	3,548	3,449 (97)	99 (3)
	UK-SCOTLAND	**UK-Scotland**	1978–2013	3,564	95.2	0.8	344	335 (97)	9 (3)
	UK-WALES	**UK-Wales**	1991–2012	959	76.1	3.1	229	229 (100)	0 (0)
**Central Europe**	AUSTRIA	**Austria**	1983–2012	2,629	96.8	4.1	623	541 (87)	82 (13)
	BELGIUM	**Belgium**	2004–2013	5,727	99.9	1.1	772	426 (55)	346 (45)
	FRANCE	Bas Rhin	1990–2014	698	99.1	1.1	100	16 (16)	84 (84)
		Basse Normandie, HM	2002–2010	994	93.1	1.5	113	5 (4)	108 (96)
		Calvados	1990–2014	42	100.0	7.1	2	2 (100)	0 (0)
		Cote dOr, HM	1990–2014	393	100.0	0.3	53	0 (0)	53 (100)
		Doubs	1990–2014	436	100.0	0.7	58	2 (3)	56 (97)
		Gironde, HM	2002–2014	884	100.0	0.2	132	3 (2)	129 (98)
		Haut-Rhin	1990–2014	511	100.0	1.6	83	24 (29)	59 (71)
		Herault	1995–2014	729	100.0	0.5	111	30 (27)	81 (73)
		Isere	1990–2014	791	100.0	0.6	108	12 (11)	96 (89)
		Loire-Atlantique/Vendée	1991–2014	1,195	100.0	0.8	195	36 (18)	159 (82)
		Manche	1994–2014	45	100.0	4.4	8	8 (100)	0 (0)
		Somme	1990–2014	435	99.8	0.7	66	10 (15)	56 (85)
		Tarn	1990–2014	264	100.0	0.4	41	7 (17)	34 (83)
	GERMANY	Bremen	2000–2013	377	98.9	0.5	51	19 (37)	32 (63)
		Common Cancer Registry of 4 Federal States^5^	2002–2013	5,493	99.1	3.1	705	442 (63)	263 (37)
		Hamburg	1998–2012	587	99.1	2.6	147	131 (89)	16 (11)
		Rhineland-Palatinate	2004–2012	1,198	93.2	2.1	198	188 (95)	10 (5)
		Saarland	1993–2012	521	99.6	1.7	77	77 (100)	0 (0)
		Schleswig-Holstein	2003–2012	1,062	94.5	1.2	158	117 (74)	41 (26)
	SWITZERLAND	Graubunden and Glarus	1989–2013	115	100.0	2.6	19	17 (89)	2 (11)
		Eastern Switzerland	1981–2013	236	100.0	2.1	51	45 (88)	6 (12)
		Ticino	2000–2012	219	100.0	1.8	33	15 (45)	18 (55)
	THE NETHERLANDS	**The Netherlands**	1989–2013	9,759	99.9	0.6	1,199	152 (13)	1047 (87)
**Southern Europe**	CROATIA	**Croatia**	2000–2012	1,178	100.0	18.1	265	265 (100)	0 (0)
	CYPRUS	**Cyprus**	2004–2014	232	100.0	3.0	38	36 (95)	2 (5)
	ITALY	Alto Adige	1995–2010	193	100.0	3.1	17	0 (0)	17 (100)
		Biella	1995–2010	191	97.9	0.5	12	10 (83)	2 (17)
		Brescia	1999–2010	290	94.1	9.3	65	65 (100)	0 (0)
		Catania-Messina-Enna	2003–2013	1,259	99.5	4.7	152	126 (83)	26 (17)
		Catanzaro	2003–2009	171	90.6	3.5	25	25 (100)	0 (0)
		Como	2003–2011	238	97.1	2.1	31	31 (100)	0 (0)
		Ferrara	1991–2011	247	100.0	2.4	26	26 (100)	0 (0)
		Friuli Venezia Giulia	1995–2010	343	100.0	3.8	75	75 (100)	0 (0)
		Genova	1986–2010	650	73.1	2.8	57	55 (96)	2 (4)
		Latina	1996–2012	308	79.5	1.9	43	37 (86)	6 (14)
		Lodi	2003–2010	129	99.2	5.4	29	28 (97)	1 (3)
		Mantova	1999–2010	123	100.0	5.7	26	26 (100)	0 (0)
		Modena	1988–2013	518	99.0	1.2	86	37 (43)	49 (57)
		Napoli	1996–2013	652	95.7	7.7	75	49 (65)	26 (35)
		Nuoro	2003–2012	114	100.0	0.0	14	14 (100)	0 (0)
		Palermo	2003–2013	712	95.2	7.0	95	94 (99)	1 (1)
		Parma	1978–2014	314	100.0	0.6	44	26 (59)	18 (41)
		Ragusa	1981–2012	375	99.7	4.3	45	44 (98)	1 (2)
		Reggio Emilia	1996–2014	407	98.8	1.0	68	30 (44)	38 (56)
		Romagna	1986–2014	934	99.0	3.5	96	87 (91)	9 (9)
		Salerno	1996–2010	571	96.1	4.9	77	76 (99)	1 (1)
		Sassari	1992–2011	209	98.6	1.4	42	42 (100)	0 (0)
		Siracusa	1999–2012	222	90.5	13.5	27	25 (93)	2 (7)
		Sondrio	1998–2013	156	84.0	4.5	20	20 (100)	0 (0)
		Trapani	2002–2010	164	100.0	2.4	33	29 (88)	4 (12)
		Trento	1995–2010	165	97.6	9.1	39	39 (100)	0 (0)
		Umbria	1994–2013	692	98.7	4.9	96	96 (100)	0 (0)
		Varese	1978–2012	348	92.2	12.9	85	83 (98)	2 (2)
		Veneto	1987–2010	1,244	96.1	2.7	147	145 (99)	2 (1)
	MALTA	**Malta**	1993–2013	192	99.0	7.8	19	19 (100)	0 (0)
	PORTUGAL	Northern Portugal	2000–2010	939	99.9	3.8	145	124 (86)	21 (14)
		Southern Portugal	2000–2012	2,055	99.9	7.8	305	262 (86)	43 (14)
	SLOVENIA	**Slovenia**	1983–2012	1,000	100.0	1.6	102	93 (91)	9 (9)
	SPAIN	Balearic Islands	1988–2012	456	99.8	1.3	65	41 (63)	24 (37)
		Basque Country	1986–2012	1,163	99.1	6.0	174	131 (75)	43 (25)
		Canarie	1996–2011	645	99.7	1.6	97	87 (90)	10 (10)
		Castellon	2004–2012	199	100.0	4.0	30	29 (97)	1 (3)
		Girona	1994–2014	475	99.8	0.4	64	14 (22)	50 (78)
		Granada	1985–2012	363	100.0	2.8	51	27 (53)	24 (47)
		Murcia	1990–2010	492	98.8	4.3	90	90 (100)	0 (0)
		Navarra	1978–2010	189	98.4	2.1	22	21 (95)	1 (5)
		Tarragona	1982–2011	336	100.0	3.0	53	35 (66)	18 (34)
**Eastern Europe**	BULGARIA	**Bulgaria**	1993–2013	2,899	100.0	8.2	690	690 (100)	0 (0)
	CZECH REPUBLIC	**Czech Republic**	1994–2013	2,975	72.2	25.8	586	468 (80)	118 (20)
	ESTONIA	**Estonia**	1978–2012	528	100.0	1.9	88	84 (95)	4 (5)
	LATVIA	**Latvia**	2000–2013	695	99.9	11.4	146	146 (100)	0 (0)
	LITHUANIA	**Lithuania**	1993–2012	2,012	99.3	3.6	325	250 (77)	75 (23)
	POLAND	**Poland**	2001–2013	8,093	95.6	9.8	2,197	2,197 (100)	0 (0)
	SLOVAKIA	**Slovakia**	1978–2010	2,067	100.0	2.0	311	257 (83)	54 (17)
**Total 84 CRs**				**106,419**	**96.1**	**4.5**	**18,083**	**14,105 (78)**	**3,978 (22)**

CML, chronic myeloid leukemia; CR, cancer registry; HM, hematological malignancies; Ph, Philadelphia chromosome.

^1^CRs period of diagnosis refers to overall data sent by each cancer registry.

^2^International Classification of Disease for Oncology, 3rd edition (ICD-O-3) codes for myeloid malignancies: 9740-9742, 9800-9801, 9805-9809, 9840, 9860-9861, 9863, 9865-9867, 9869-9876, 9891, 9895-9898, 9910-9911, 9920, 9930-9931, 9945-9946, 9950, 9960-9964, 9966, 9975, 9980, 9982-9987, 9989, 9991-9992.

^3^ICD-O-3 codes of CML cases eligible for the survival analysis: 9863 (CML with no cytogenetic information, CML NOS) and 9875 (Ph+, BCR/ABL1-positive CML).

^4^Myeloid NOS cases ICD-O-3 codes: 9800, 9801, 9805, and 9860.

^5^Four Federal States: Brandenburg, Mecklenburg-Western Pomerania, and the Free States of Saxony and Thuringia.

CRs with national coverage are in bold.

The EUROCARE-6 patient complete selection is reported in the **Supplementary Material**.

### 2.2 Statistical Methods

#### 2.2.1 RCT Meta-Analysis Data

OS data by follow-up time, number of deaths and hazard ratios (HRs), and cancer-specific mortality were collected through the RCTs included in the published meta-analysis (58, 59).

The OS data were pooled using the inverse variance method. Study heterogeneity was evaluated by calculating the I-squared statistic (I^2^) with little, moderate, and substantial heterogeneity being indicated by I^2^ values of <50%, 50%–75%, and >75%, respectively. Ninety-five percent confidence intervals (CIs) and two-sided p-values were calculated for each result.

#### 2.2.2 Population-Based Data

Five-year crude OS of CML cases (9863, 9875 ICD-O-3 codes), aged between 15 and 64, diagnosed in 2000–2006 and 2007–2013, by European region and country, was estimated from the EUROCARE-6 study dataset. The 64-year threshold was determined, considering CML RCTs inclusion criteria and to make the age of patients more comparable between RCTs (median age: 50 years; range: 18–91) (**Table 1**) (58, 59) and population-based EUROCARE-6 results (median age: 50 years) (**Table 3**). The period of diagnosis threshold (pre- and post-2006) was established considering the timing of second-generation TKIs introduction (dasatinib and nilotinib) in clinical practice.

**Table 3:** Five-year crude overall survival of CML cases (15–64 years) (9863, 9875 ICD-O-3 codes)^1^ diagnosed in 2000–2006 and 2007–2013 by European region and country. EUROCARE-6 study dataset.

Country/Area	Total cases 2000–2013	Median age (years)	Male	M %	2000–2006						2007–2013					Absolute difference	p-value
					N at start	N_5_	OS	95%CI		N at start		N_5_	OS	95%CI			
**Northern Europe (4 CRS)**	**1,109**	**48**	**621**	**56.0**	**534**	**438**	**80.5**	**77.2**	**83.9**	**575**		**314**	**89.2**	**86.4**	**92.2**	8.8**	**<0.001**
Denmark	470	48	267	56.8	225	186	80.8	75.8	86.1	245		135	88.7	84.0	93.6	7.9*	**0.028**
Finland	304	49	175	57.6	165	135	80.0	74.1	86.3	139		73	86.0	79.8	92.6	6.0	0.187
Iceland	23	45	18	78.3	11	9	–	–	–	12		6	–	–	–	–	–
Norway	312	48	161	51.6	133	108	80.5	74.0	87.5	179		100	91.8	87.7	96.0	11.3**	**0.005**
**UK and Ireland (4 CRs)**	**4,361**	**49**	**2,555**	**58.6**	**2,001**	**1,488**	**72.2**	**70.3**	**74.2**	**2,360**		**1,187**	**86.9**	**85.3**	**88.4**	14.7**	**<0.001**
Ireland	240	52	141	58.8	117	97	79.5	72.5	87.2	123		58	90.7	85.3	96.5	11.2*	**0.017**
England	3548	48	2080	58.6	1596	1167	70.9	68.7	73.2	1952		982	86.5	84.8	88.2	15.5**	**<0.001**
Scotland	344	50	210	61.0	166	139	83.1	77.6	89.0	178		87	87.8	82.1	93.7	4.6	0.263
Wales	229	50	124	54.1	122	85	67.2	59.4	76.1	107		60	88.2	81.7	95.2	21.0**	**<0.001**
**Central Europe (25 CRs)**	**5,103**	**50**	**2,958**	**58.0**	**2,186**	**1,829**	**82.6**	**81.0**	**84.2**	**2,917**		**1,407**	**88.5**	**87.1**	**89.9**	5.9**	**<0.001**
Austria	623	51	379	60.8	347	262	74.6	70.2	79.4	276		146	84.2	79.6	89.1	9.5**	**0.005**
Belgium	772	50	437	56.6	201	176	87.0	82.5	91.8	571		282	92.0	89.5	94.6	5.0	0.066
France (13 CRs Pool)	1070	50	628	58.7	444	394	88.5	85.6	91.5	626		321	92.1	89.5	94.7	3.6	0.076
Germany (6 CRs Pool)	1336	50	786	58.8	597	502	82.6	79.6	85.7	739		312	85.7	82.7	88.8	3.2	0.150
Switzerland (3CRs Pool)	103	50	60	58.3	51	47	90.2	82.4	98.7	52		26	85.8	74.4	99.0	−4.4	0.561
The Netherlands	1199	49	668	55.7	546	448	80.4	77.1	83.8	653		320	87.2	84.2	90.2	6.7**	**0.003**
**Southern Europe (44 CRs)**	**3,167**	**49**	**1,855**	**58.6**	**1,738**	**1,396**	**78.1**	**76.2**	**80.1**	**1,429**		**816**	**86.9**	**85.0**	**88.8**	8.8**	**<0.001**
Cyprus	38	49	28	73.7	10	9	–	–	–	28		19	–	–	–	–	–
Croatia	265	52	166	62.6	154	100	59.7	52.5	68.0	111		25	68.6	57.7	81.5	8.8	0.220
Italy (29 CRs Pool)	1647	50	950	57.7	906	752	81.3	78.8	83.9	741		441	88.3	85.8	90.8	7.0**	**<0.001**
Malta	19	40	12	63.2	12	9	–	–	–	7		2	–	–	–	–	–
Portugal (2 CRs Pool)	450	49	253	56.2	254	191	74.0	68.8	79.6	196		121	82.9	77.5	88.6	8.9*	**0.025**
Slovenia	102	49	65	63.7	54	35	59.3	47.5	73.9	48		30	91.7	84.2	99.8	32.4**	**<0.001**
Spain (CRs Pool)	646	47	381	59.0	348	300	83.6	79.8	87.6	298		178	90.3	86.7	94.0	6.7*	**0.014**
**Eastern Europe (7 CRs)**	**4,343**	**51**	**2,376**	**54.7**	**2,334**	**1,351**	**55.3**	**53.3**	**57.3**	**2,009**		**754**	**72.8**	**70.6**	**75.1**	17.6**	**<0.001**
Bulgaria	690	53	374	54.2	390	174	41.3	36.7	46.5	300		106	63.6	58.0	69.7	22.3**	**<0.001**
Czech Republic	586	50	329	56.1	336	228	66.1	61.2	71.3	250		81	75.0	68.5	82.2	9.0*	**0.039**
Estonia	88	50	54	61.4	53	30	54.7	42.8	69.9	35		19	69.0	54.5	87.3	14.2	0.186
Latvia	146	50	82	56.2	67	40	56.7	46.0	69.9	79		28	63.8	52.8	77.1	7.1	0.414
Lithuania	325	49	173	53.2	179	92	49.1	42.3	57.0	146		73	78.7	71.9	86.1	29.5**	**<0.001**
Poland	2197	50	1195	54.4	1105	669	57.8	55.0	60.8	1092		384	74.3	71.2	77.6	16.5**	**<0.001**
Slovakia	311	50	169	54.3	204	118	55.4	49.0	62.7	107		63	78.2	70.3	86.9	22.8**	**<0.001**
**European Pool (84 CRs)**	**18,083**	**50**	**10,365**	**57.3**	**8,793**	**6,502**	**71.9**	**71.0**	**72.9**	**9,290**		**4478**	**84.7**	**83.9**	**85.5**	12.7**	**<0.001**

CI, confidence interval; CML, chronic myeloid leukemia; CR, cancer registry; ICD-O-3, International Classification of Disease for Oncology, 3rd edition; M, male; N at start, number of CML cases alive at the beginning of the period; N_5_, number of CML cases alive at 5 years from diagnosis; OS, overall survival.

^1^ICD-O-3 codes of CML cases eligible for the survival analysis: 9863 (CML with no cytogenetic information, CML NOS) and 9875 (Ph+, BCR/ABL1-positive CML).

Survival estimates are not provided for strata including fewer than 10 cases.

**p-value <0.01 and *p-value <0.05.

In bold European regions and statistically significant p values.

As most CRs do not collect data concerning disease phase, we used conditional survival (63) to select patients who are potentially in the CP, thus excluding the short-term mortality associated with BP or AP CML. Therefore, conditional crude OS (i.e., the probability of being alive after 5 years, conditional on surviving 3 years after diagnosis, in brief 5-/3-year OS ratio) was computed on the assumption that patients with CML surviving more than 3 years are not likely to include patients in AP and BP.

Relative survival (RS) (64), defined as OS divided by the expected survival of a comparable group (i.e., of the same age, sex and area) from the general population not affected by CML, was estimated using the complete approach (65). Expected survival was estimated using the Ederer II method (66). Conditional crude RS was computed in terms of 5-/3-year RS ratio.

Standard errors (SEs) of OS and RS were derived by applying Greenwood’s formula (67). SE for conditional survival were calculated with the delta method (63). To obtain two-sided 95% CIs, the data were logarithmically transformed. The statistical significance of survival differences between patients diagnosed before and after 2006 (2000–2006 vs. 2007–2013) was tested with the Z-test (68).

#### 2.2.3 Comparison Between RCTs and Population-Based Survival

We compared both OS, including all causes of death for patients with CML, and RS, a proxy of cause-specific survival, i.e., discarding competitive causes of mortality other than CML. Because, for RCTs, RS is not available (as they record the specific cause of death), we estimated the 5-year cause-specific survival (i.e., “freedom from death due to advanced CML”) using data extracted from the corresponding RCTs included in the meta-analysis (58, 59).

The analyses were made using Review Manager v. 5.3 and SEER*Stat software 8.3.9.

## 3 Results

### 3.1 RCTs Results

Many of the RCTs did not report OS at each and every one of the time points, but the patients were closely followed-up (**Table 1**). Only two RCTs reported OS up to 60 months (data not pooled), and only one reported OS up to 72 months. Five-year OS in the ENESTnd (38, 45) study was similar in the imatinib and nilotinib groups [92% vs. 94% for nilotinib of 300 mg (HR = 0.80; 95% CI, 0.43–1.50), and 96% for nilotinib of 400 mg (HR = 0.44; 95% CI, 0.21–0.93)]. Similar results were obtained in the DASISION (23, 32) study comparing imatinib with dasatinib: 5-year OS 90% vs. 91% (HR = 1.01; 95% CI, 0.58–1.73). The first follow-up time point at which it was possible to analyse pooled OS was 36 months (data from three RCTs), but, as it was not clinically relevant, we pooled the HRs roughly extracted from the printed OS curves of Radich et al. (33) (36-month of follow-up) and the ENESTnd (38, 45) and DASISION (23, 32) HRs (60-month follow-up) on the basis of the proportional hazards assumption; the result was not statistically significant (OS: HR = 0.78; 95% CI, 0.54–1.11) (58, 69).

The BFORE study update showed that 5-year OS was similar between bosutinib and imatinib (95% vs. 95%; HR = 0.95; 95% CI, 0.45–1.99) (56).

### 3.2 EUROCARE-6 Results

The numbers of patients with CML eligible for the survival analysis are reported by CR (**Table 2**). The main characteristics of patients included in survival analysis and the 5-year crude OS values of all CML cases (9863 - CML NOS, 9875 - Ph+ CML ICD-O-3 codes) are shown by European region and country (**Table 3**). The 9875 - Ph+ CML ICD-O-3 code is scarcely adopted (22%) (**Table 2**).

Comparing OS results between the two periods of diagnosis (2000–2006 vs. 2007–2013), a clear increase of OS values was observed for all European regions and for most countries (**Table 3**). A marked statistically significant increase was observed in the pool of all European countries (71.9% for patients diagnosed in 2000–2006 vs. 84.7% diagnosed in 2007–2013; absolute difference: 12.7%) and in all European areas, with higher improvements (>10%) in Eastern Europe (17.6%) and United Kingdom and Ireland (14.7%). Considering each country, the highest significant increases (>20%) were observed for Wales (21.0%), Slovenia (32.4%), Bulgaria (22.3%), Lithuania (29.5%), and Slovakia (22.8%). Notably, in most Western European countries, OS of patients diagnosed in 2007–2013 was similar to CP CML OS reported in RCTs (**Table 1**).

The study evaluated crude 5-/3-year conditional OS of all CML cases (i.e., the probability of being alive after 5 years, conditional on surviving 3 years after diagnosis), likely representing patients with CML in CP, diagnosed in 2000–2006 and 2007–2013, by European region and country (**Table 4**). A significant increase was observed in Europe as a whole (92.9% in 2000–2006 vs. 96.1% in 2007–2013; absolute difference: 3.2%) and in all areas except in Northern and Central Europe, showing that the most substantial 5-year OS increase (12.7%, **Table 3**) was concentrated in the first 3-year prognosis. Notably, countries with more marked delta OS increases (Slovenia, Lithuania, Bulgaria, and Slovakia; **Table 3**) showed the highest growth even in the CP (**Table 4**). Time trends of crude 5-/3-year conditional RS of all CML cases are presented in **Supplementary Table 4**. Conditional RS values are slightly higher than conditional OS values (by 1.1% on average), reflecting the limited impact of excluding causes of death other than CML in patients aged under 65 at diagnosis. Time trends of conditional RS are quite similar to those estimated for conditional OS. Small significant overall increases were estimated in the European pool (94.0% in 2000–2006 vs. 97.2% in 2007–2013; absolute difference: 3.2%) and in all areas but Northern and Central Europe.

**Table 4:** Conditional crude 5-/3-year overall survival^1^ of CML cases (15–64 years) (9863, 9875 ICD-O-3 codes)^2^ diagnosed in 2000–2006 and 2007–2013 by European region and country. EUROCARE-6 study dataset.

Country/Area	2000–2006					2007–2013					Absolute difference	p-value
	N_3_	N_5_	5-/3-year	95%CI		N_3_	N_5_	5-/3-year	95%CI			
**Northern Europe (4 CRS)**	**470**	**438**	**95.7**	**93.9**	**97.6**	**475**	**314**	**96.4**	**94.3**	**98.5**	0.7	0.642
Denmark	199	186	94.2	91.0	97.6	199	135	93.8	89.7	98.1	−0.4	0.873
Finland	143	135	96.4	93.3	99.5	114	73	96.0	91.7	100.6	−0.3	0.907
Iceland	9	9	–	–	–	10	6	–	–	–	–	–
Norway	119	108	97.3	94.3	100.4	152	100	100.0	100.0	100.0	2.7	0.079
**UK and Ireland (4 CRs)**	**1,641**	**1,488**	**92.9**	**91.6**	**94.2**	**1,872**	**1,187**	**97.2**	**96.2**	**98.1**	4.3**	**<0.001**
Ireland	105	97	93.0	88.1	98.1	99	58	98.6	95.8	101.4	5.6	0.057
England	1293	1167	92.6	91.1	94.0	1537	982	97.3	96.3	98.4	4.8**	**<0.001**
Scotland	146	139	94.5	90.9	98.3	144	87	95.1	90.6	99.9	0.6	0.832
Wales	97	85	94.3	89.5	99.3	92	60	96.6	92.0	101.4	2.3	0.497
**Central Europe (25 CRs)**	**1,937**	**1,829**	**96.3**	**95.4**	**97.1**	**2,372**	**1,407**	**96.0**	**95.0**	**97.0**	−0.2	0.719
Austria	280	262	96.3	94.0	98.6	252	146	93.9	90.2	97.7	−2.4	0.274
Belgium	185	176	96.7	94.1	99.3	471	282	98.2	96.5	99.8	1.5	0.344
France (13 CRs Pool)	415	394	97.3	95.7	98.9	523	321	96.6	94.6	98.6	−0.7	0.588
Germany (6 CRs Pool)	529	502	96.3	94.7	97.9	545	312	95.2	92.9	97.5	−1.1	0.453
Switzerland (3 CRs Pool)	50	47	95.8	90.3	101.7	42	26	91.9	81.5	103.6	−3.9	0.535
The Netherlands	478	448	95.2	93.3	97.2	539	320	95.7	93.6	97.9	0.5	0.725
**Southern Europe (44 CRs)**	**1,509**	**1,396**	**94.3**	**93.2**	**95.5**	**1,209**	**816**	**97.2**	**96.1**	**98.4**	2.9**	**0.001**
Cyprus	10	9	–	–	–	27	19	–	–	–	–	–
Croatia	116	100	87.6	81.5	94.2	59	25	91.9	81.5	103.7	4.3	0.510
Italy (29 CRs Pool)	799	752	95.3	93.8	96.8	624	441	97.6	96.2	99.1	2.3*	**0.028**
Malta	10	9	–	–	–	3	2	–	–	–	–	–
Portugal (2 CRs Pool)	219	191	93.5	90.2	97.0	173	121	96.0	92.7	99.5	2.5	0.301
Slovenia	41	35	86.5	76.1	98.2	44	30	100.0	100.0	100.0	13.5*	**0.016**
Spain (CRs Pool)	314	300	95.7	93.5	98.0	279	178	97.4	95.1	99.7	1.7	0.313
**Eastern Europe (7 CRs)**	**1,636**	**1,351**	**86.6**	**84.9**	**88.4**	**1,295**	**754**	**93.4**	**91.6**	**95.1**	6.7**	**<0.001**
Bulgaria	241	174	78.2	72.7	84.0	195	106	95.1	91.3	99.0	17.0**	**<0.001**
Czech Republic	256	228	92.5	89.2	95.9	144	81	93.5	88.0	99.2	1.0	0.771
Estonia	39	30	80.6	68.6	94.6	29	19	86.4	73.1	102.0	5.8	0.556
Latvia	49	40	82.6	72.4	94.3	52	28	92.7	83.3	103.2	10.1	0.180
Lithuania	121	92	82.2	75.3	89.8	122	73	95.0	90.4	99.9	12.8**	**0.004**
Poland	791	669	88.0	85.7	90.4	665	384	92.6	90.1	95.2	4.6**	**0.010**
Slovakia	139	118	88.3	82.9	94.0	88	63	95.0	89.6	100.7	6.7	0.093
**European Pool (84 CRs)**	**7,193**	**6,502**	**92.9**	**92.3**	**93.5**	**7,223**	**4,478**	**96.1**	**95.5**	**96.7**	3.2**	**<0.001**

CI, confidence interval; CML, chronic myeloid leukemia; CR, cancer registry; ICD-O-3, International Classification of Disease for Oncology, 3rd edition.

N_3_ and N_5_, number of CML cases alive at 3 and 5 years from diagnosis, respectively.

^1^The crude 5-/3-year conditional overall survival is the probability of being alive after 5 years, conditional on surviving 3 years after diagnosis.

^2^ICD-O-3 codes of CML cases eligible for the survival analysis: 9863 (CML with no cytogenetic information, CML NOS) and 9875 (Ph+, BCR/ABL1-positive CML).

Survival estimates are not provided for strata including fewer than 10 cases.

**p-value <0.01 and *p-value <0.05.

In bold European regions and statistically significant p values.

In **Supplementary Tables 2, 3** were reported 5-year crude OS and 5-year crude RS, respectively, of CML cases diagnosed in 2000–2006 and 2007–2013 by European region, country, and morphology code. The differences between OS and RS were small, probably due to the patients’ age selection (15–64 years, with negligible competitive mortality). In particular, in **Supplementary Table 2**, were compared OS values between 9863 CML NOS and 9875 Ph+ CML codes in 2000–2006 and 2007–2013, by areas: in all areas, CML NOS cases showed a lower OS values in comparison with Ph+ CML, even if differences reduced over time (except for Eastern Europe).

### 3.3 Comparisons Between RCTs and EUROCARE-6 Results

The estimated values of 5-year cause-specific survival in the ENESTnd study were 97.7% (96.0–99.5%) for nilotinib of 300 mg, 98.5% (97.1–100.0%) for nilotinib 400 mg, 93.8% (90.8–96.7%) for imatinib of 400 mg (38, 45). The DASISION study (23, 32) only reported the number of patients who had died of CML-related causes after 5 years of follow-up: 17/260 in the imatinib arm and 9/259 in the dasatinib arm. The estimated values of 5-year cause-specific survival in CP CML RCTs (58, 59) were quite similar to 5-/3-year conditional crude RS of all CML cases estimated in the best ranking countries of the EUROCARE-6 dataset. They are also close to the 5-/3-year conditional crude RS estimates for the European pool (97.2% in 2007–2013) (**Supplementary Table 4**).

## 4 Discussion

The comparison of EUROCARE-6 CML survival estimated in patients diagnosed in 2000–2006 vs. 2007–2013 confirmed that the prognostic improvement highlighted by RCTs was verifiable in real-world settings. In particular, the EUROCARE-6 OS values in many countries (**Table 3**) were very similar to CP CML OS reported in RCTs (**Table 1**) (58, 59). Moreover, the same brilliant achievement was observed comparing the estimated values of 5-year cause-specific survival in CP CML RCTs (58, 59) with 5-/3-year conditional crude RS estimated in almost all European countries in 2007–2013 (**Supplementary Table 4**). This means that the optimal outcome figures obtained in controlled settings are achievable (and, indeed, are achieved) in real-world settings, too. The high concordance between CRs and RCTs survival results could be explained by the fact that TKIs are responsible of the quite complete disappearance of AP and BP worse prognosis CML phases. Almost all patients are diagnosed in CP (or have been quickly brought back to CP), so survival results reported in the whole population are close to those of RCTs. Moreover, the high concordance between CRs and RCTs survival results could be related to the fact that we compared quite homogeneous groups of patients with CML aged lower than 65 years with probably few comorbidities.

Previous population studies reported similar or inferior survival results but estimated only on national or small pooled samples.

Swedish CML Registry (779 CMLs, from 2002 to 2010; median age, follow-up: 60 years, 61 months) showed 5-year RS close to 1.0 for those younger than 60 years, 0.9 for those aged 60 to 80 years, and 0.6 for those older than 80 years (70). Swedish Cancer Registry (2,662 CMLs, from 1973 to 2013; median age: 69 years) reported clear improvements in life expectancy over the study period (71). Swedish Cancer Registry and Swedish Cause of Death Registry (CMLs, from 1970 to 2012) showed 5-year OS increasing from 0.18 to 0.82, during the study period; between 2006 and 2012, 5-year RS was close to normal for 40-year-old but considerably lower for 80-year-old patients (72). UK’s Haematological Malignancy Research Network (242 CMLs, from 2004 to 2011; median age: 59 years) showed 5-year OS of 78.9% (72.3% to 84.0%) and 5-year RS of 88.6% (81.0% to 93.3%) (73). Other national studies are aligned with our survival results (74–80).

European Treatment and Outcome Study (EUTOS) (2,904 CMLs, from 2008 to 2013; median age, follow-up: 55 years, 29 months) showed a 30 months OS of 92% (81). US Surveillance, Epidemiology, and End Results (SEER) (13,869 CMLs, from 1975 to 2009) reported lower survival values: 5-year RS ratios increased from 0.26 in 1975–1989 to 0.36 in 1990–2000 and 0.56 in 2001–2009 (82). Moreover, SEER (5,138 CMLs, from 2000 to 2005) showed 5-year OS improvement for all patients during the study period (83, 84). Compared with patients diagnosed in 2000, 5-year OS improved among 15–44 years (from 71.6% to 86.4%), 45–64 years (from 67.5% to 76.3%), 65–74 years (from 38.1% to 51.2%), and 75–84 years patients (from 19.2% to 36.4%) (83).

Population-based studies using real-world survival data reveal differences from the values observed in RCTs that are often related to treatment disparities and largely due to different socioeconomic conditions. They also provide information concerning treatment effectiveness in everyday clinical practice without any patient or outcome selection: they are therefore more representative of what happens in real-life, despite lacking in clinical details offered by RCTs, particularly in relation to disease stage at the time of diagnosis and first-line treatments. The findings of RCTs are often used to guide clinical practice (particularly in oncology), but patient selection can reduce their applicability to the general population (17, 18, 20, 21). Conversely, results of population-based CR studies that fully cover the target population are less affected by patient selection biases, and they provide useful data complementing RCTs outcomes.

However, these two information sources need to be integrated and require the use of new study designs and methods of analysis. High-resolution population-based studies, which include representative patients, present more detailed clinical information than that which is routinely collected by population-based CRs: this approach may help to reduce the gap between RCTs and real-world studies (hrstudies.it; https://www.ipaac.eu/en/work-packages/wp7/).

In an attempt to quantify the difference between RCTs and population-based studies using tangible data, we compared OS and cause-specific survival observed in the RCTs included in our previous systematic review (58), and OS and RS values estimated using EUROCARE-6 (22) cases diagnosed up to age 64 over a comparable period of time. It was the first time that this was done for CML, considering all European regions and pooling survival results. Our study shows that CML survival values tend to become very similar between RCTs and population-based settings, regardless of the survival analysis methods used. However, some differences still persist, in particular in Eastern European countries, where OS values were lower than elsewhere, especially in the first period of time being considered: this is probably due to a delayed introduction of TKIs in daily clinical practice. To underline that the date of the introduction of TKIs reimbursement varied greatly between Europe: this could be useful to interpret the different survival outcomes observed by countries (**Supplementary Table 5**). Also to notice that the allogeneic bone marrow transplantations medium rate was 0.62 per million for Eastern European countries in comparison with 0.81 per million for other European countries [**Supplementary Table 6**, by calendar year from 2000 to 2022 and by country; data provided by the European Society for Blood and Marrow Transplantation (EBMT), Chronic Malignancies Working Party (CMWP)].

Residual discrepancies can be attributed to different case selection criteria: RCTs select patients on the basis of well-defined inclusion and exclusion criteria, and the results cannot be readily extended to the general population, whereas population-based studies involve unselected patients but often lack detail and, in the case of CML, the morphology code might be not very precise. Moreover, RCTs almost always record cancer-specific mortality, with off-study survival being reported by the investigator after study discontinuation, whereas population studies systematically update life status of all registered patients and use RS to make adjustments for general mortality by age, gender, and geographical area.

RCTs also generally include patients without comorbidities who are younger than those encountered in real-life populations: for example, it has been found that the elderly, women, and members of racial and ethnic minorities are less likely to be enrolled in American cooperative group cancer trials than patients who are younger, male, and Caucasian (85, 86).

Our previous meta-analysis did not reveal any difference in the OS of patients treated with the first- or the new-generation TKIs (58, 59). In the only two RCTs for which 5-year OS data are available [DASISION (23, 32) and ENESTnd (38, 45)], the 60-month OS value was similar in the patients treated with imatinib and those treated with dasatinib or nilotinib, and similar to EUROCARE-6 OS data for patients diagnosed in 2007–2013. To underline that second-generation TKIs introduction time in clinical practice (2006–2007) limits a strict comparison with survival data of previous years, but imatinib can be considered an historical arm because it has been introduced in 2001. Moreover, CML survival values under imatinib or second-generation TKIs are fairly superimposable (60 months RCTs OS ≥ 90%, **Table 1**).

We compared the first-line treatment of RCT patients with newly diagnosed CP CML with all treatment lines administered to patients with CML from the general population (including a small percentage of patients with AP and BP CML who have a different prognosis). Unfortunately, CRs do not routinely collect information on CML phase and treatment line; thus, it was not possible to select CP CML cases receiving first-line treatment. To overcome this drawback, we analyzed 5-/3-year conditional OS and RS to remove the contribution of BP and AP CML and improve estimates comparability. Considering conditional OS and RS for patients diagnosed in 2007–2013, population-based CRs survival values were very similar to those observed in the RCTs.

Code 9876 (Ph− atypical CML or aCML) was not included but, as most CRs do not distinguish Ph+ CML and Ph− aCML, and as 78.0% of cases are classified as CML NOS (**Table 2**), some aCML cases were inevitably included. This has little impact on our analysis as 90%–95% of CML diagnoses have the characteristic t(9;22)(q34;q11.2) reciprocal translocation, leading to the Ph chromosome and to the *BCR-ABL1* fusion gene that is the target for specific TKIs (4). However, this partly explains why OS values for ICD-O-3 code 9863, including CML NOS and (probably) patients with poorer prognosis (such as aCML cases not targeted by TKIs), were, at all evaluable times and in all evaluable regions, lower compared to the values for Ph+ CML for which TKIs are indicated.

Code 9875 (Ph+ CML) was hardly used in Northern Europe or the United Kingdom and Ireland, and the implausibly small number of cases in the other regions/countries considered is attributable to differences in registration criteria or inaccurate pathological description. It is also likely that the underuse of code 9875 for Ph+ CML is due to a bad translation of the ICD-O-3 classification: code 9863 refers to “chronic myeloid leukemia, NOS” and code 9875 refers to “chronic myelogenous leukemia, BCR/ABL positive” (Ph+ CML) and, although hematologists normally correctly diagnose cases of code 9875 as Ph+ CML, the use of the word “myelogenous” is ambiguous for non-hematologists. This may also explain the considerable difference in the use of code 9875 between specialized hematological registries and general CRs. CRs should correctly code CML morphology by specifying ICD-O-3 9875 (Ph+ CML) or 9876 (Ph− aCML), the phase of the disease at the time of diagnosis, first-line therapy, and the occurrence of transformation into AP or BP to make a more precise analysis possible: one that is potentially comparable with other types of studies. Some strategies should be adopted to avoid CML code misuse and to reduce the number of CML NOS cases, such as to plan specific training courses to increase the precision of coding or to link CML population-based data with other available data sources, for example, national health insurance databases, to discover patients really treated with TKIs (87). Unfortunately, 9875 (Ph+ CML) code is so underused in CRs in the studied period 2000–2013 not to permit to design a population-based study excluding 9863 code (CML NOS).

A clear improvement in real-world CML survival was observed in European regions and countries comparing EUROCARE-6 with RCTs OS data. However, some discrepancies with RCTs still remain. Our results suggest an insufficient adoption of adequate protocols in daily clinical practice in countries where CML survival values still remain lower in real-life than those obtained in RCTs. In future works, it will be of interest to focus on populations usually excluded from RCTs, such as older patients, or with comorbidities and other cancers.

## EUROCARE-6 Working Group

**Austria:** M. Hackl (*National CR*); **Belgium**: E. Van Eycken (*National CR*); **Bulgaria**: Z. Valerianova (*National CR*); **Croatia**: M. Sekerija (*National CR*); **Cyprus**: P. Pavlou (*National CR*); **Czech Republic**: L. Dušek (*National CR*); **Denmark**: H. Storm (*National CR*); **Estonia**: M. Mägi; K. Innos* (*National CR*); **Finland**: N. Malila; J. Pitkäniemi (*National CR*); **France**: M. Velten (*Bas Rhin CR*); X. Troussard (*Basse Normandie*, *Haematological Malignancies CR*); A.M. Bouvier; V. Jooste* (*Burgundy*, *Digestive CR*); A.V. Guizard (*Calvados*, *General CR*); G. Launoy (*Calvados*, *Digestive CR*); S. Dabakuyo Yonli (*Cote dOr*, *Gynaecologic* (*Breast*) CR); M. Maynadié (*Cote dOr*, *Haematological Malignancies CR*); A.S. Woronoff (*Doubs CR*); J.B. Nousbaum (*Finistere*, *Digestive CR*); G. Coureau (*Gironde*, *General CR*); A. Monnereau* (*Gironde*, *Haematological Malignancies CR*); I. Baldi (*Gironde*, *Central Nervous System CR*); K. Hammas (*Haut-Rhin CR*); B. Tretarre (*Herault CR*); M. Colonna (*Isere CR*); S. Plouvier (*Lille Area CR*); T. D’Almeida (*Limousin CR*); F. Molinié; A. Cowppli-Bony (*Loire-Atlantique/Vendée CR*); S. Bara (*Manche CR*); C. Schvartz (*Marne-Ardennes*, *Thyroid CR*); G. Defossez (*Poitou-Charentes CR*); B. Lapôtre-Ledoux (*Somme CR*); P. Grosclaude (*Tarn CR*); **Germany**: S. Luttmann (*Bremen CR*); R. Stabenow [*Common CR of 4 Federal States* (*Brandenburg*, *Mecklenburg-West Pomerania*, *Saxony-Anhalt*, *Thüringen*)]; A. Nennecke (*Hamburg CR*); J. Kieschke (*Lower Saxony CR*); S. Zeissig (*Rhineland-Palatinate CR*); B. Holleczek (*Saarland CR*); A. Katalinic* (*Schleswig-Holstein CR*); **Iceland**: H. Birgisson (*National CR*); **Ireland**: D. Murray; P.M. Walsh (*National CR*); **Italy**: G. Mazzoleni; F. Vittadello (*Alto Adige CR*); F. Cuccaro (*Barletta-Andria-Trani CR*); R. Galasso (*Basilicata CR*); G. Sampietro (*Bergamo CR*); S. Rosso (*Biella CR*); M. Magoni (*Brescia CR*); M. Ferrante (*Catania-Messina-Enna CR*); A. Sutera Sardo (*Catanzaro CR*); M.L. Gambino (*Como CR*); P. Ballotari; E. Giacomazzi (*Cremona and Mantova CR*); S. Ferretti (*Ferrara CR*); A. Caldarella; G. Manneschi (*Firenze-Prato CR*); G. Gatta*; M. Sant*; P. Baili*; F. Berrino*; L. Botta; A. Trama; R. Lillini; A. Bernasconi; S. Bonfarnuzzo; C. Vener; F. Didonè; P. Lasalvia; G. Del Monego; M.C. Magri; L. Buratti (*Fondazione IRCCS Istituto Nazionale dei Tumori*, *Milan*); D. Serraino; L. Dal Maso (*Friuli Venezia Giulia CR*); R. Capocaccia* (*Epidemiologia e Prevenzione Board*); R. De Angelis*; E. Demuru; C. Di Benedetto; S. Rossi*; M. Santaquilani; S. Venanzi (*Istituto Superiore di Sanità*, *Rome*); R.A. Filiberti (*Genova CR*); S. Iacovacci (*Latina CR*); V. Gennaro (*Liguria*, *mesotheliomas CR*); A.G. Russo (*Lodi CR*); G. Spagnoli (*Modena CR*); L. Cavalieri d’Oro (*Monza and Brianza CR*); M. Fusco; M.F. Vitale (*Napoli CR*); M. Usala (*Nuoro CR*); F. Vitale (*Palermo CR*); M. Michiara (*Parma CR*); G. Chiranda (*Piacenza CR*); G. Cascone; E. Spata (*Ragusa CR*); L. Mangone (*Reggio Emilia CR*); F. Falcini (*Romagna CR*); R. Cavallo (*Salerno CR*); D. Piras (*Sassari CR*); A. Madeddu; F. Bella (*Siracusa CR*); A.C. Fanetti (*Sondrio CR*); S. Minerba (*Taranto CR*); G. Candela; T. Scuderi (*Trapani CR*); R.V. Rizzello (*Trento CR*); F. Stracci (*Umbria CR*); G. Tagliabue (*Varese CR*); M. Rugge (*Veneto CR*); A. Brustolin (*Viterbo CR*); **Latvia**: S. Pildava (*National CR*); **Lithuania**: G. Smailyte (*National CR*); **Malta**: M. Azzopardi (*National CR*); **Norway**: T.B. Johannesen* (*National CR*); **Poland**: J. Didkowska; U. Wojciechowska (*National CR*); M. Bielska-Lasota* (*National Institute of Public Health-National Institute of Hygiene-National Research Institute*, *Warsaw*); **Portugal**: A. Pais (*Central Portugal CR*); J.L. Pontes (*Northern Portugal CR*); A. Miranda (*Southern Portugal CR*); **Slovakia**: C. Safaei Diba (*National CR*); **Slovenia**: V. Zadnik; T. Zagar (*National CR*); **Spain**: C. Sánchez-Contador Escudero; P. Franch Sureda (*Balearic Islands*, *Mallorca CR*); A. Lopez de Munain; M. De-La-Cruz (*Basque Country CR*); M.D. Rojas, A. Aleman (*Canary Islands CR*); A. Vizcaino (*Castellon CR*); R. Marcos-Gragera (*Girona CR*); M.J. Sanchez (*Granada CR*); M.D. Chirlaque (*Murcia CR*); M. Guevara Eslava*; E. Ardanaz (*Navarra CR*); J. Galceran; M. Carulla (*Tarragona CR*); **Switzerland**: Y. Bergeron (*Fribourg CR*); C. Bouchardy (*Geneva CR*); S. Mohsen Mousavi (*Graubünden and Glarus CR*); S. Mohsen Mousavi (*Eastern Switzerland CR*); A. Bordoni (*Ticino CR*); **The Netherlands**: O. Visser* (*National CR*); **UK-England**: J. Rashbass (*National CR*); **UK-Northern Ireland**: A. Gavin* (*National CR*); **UK-Scotland**: D. Morrison (*National CR*); **UK-Wales**: D. W. Huws* (*National CR*).*EUROCARE Steering Committee

## Data Availability Statement

The datasets presented in this article are not readily available. See EUROCARE-6 Collaborative Group Rules (http://www.eurocare.it). Requests to access the datasets should be directed to http://www.eurocare.it.

## Ethics Statement

Ethical review and approval was not required for the study on human participants in accordance with the local legislation and institutional requirements. Written informed consent from the participants’ legal guardian/next of kin was not required to participate in this study in accordance with the national legislation and the institutional requirements.

## Author Contributions

CV designed and carried out the study and analyzed the data; SR and PM did quality controls and analyzed the data; RA and MS designed the study and data quality checks; RM-G, HP, MM, XT, and GP provided advice and revised the results. EUROCARE-6 Working Group collected, prepared, and transmitted raw data for the study database; corrected data after quality controls; and checked the results of the analyses. All authors contributed to the article and approved the submitted version.

## Funding

This study was funded by the Compagnia di San Paolo, the Cariplo Foundation and the European Commission (grant number 801520 HP-JA-2017, Innovative Partnership for Action Against Cancer, iPAAC Joint Action). The sources of the funding played no role in designing the study, collecting, analyzing, or interpreting the data, writing the report, or deciding whether or not to submit the article for publication. This research was (partially) funded by Italian Ministry of Health “Ricerca Corrente” funds.

## Conflict of Interest

The authors declare that the research was conducted in the absence of any commercial or financial relationships that could be construed as a potential conflict of interest. 

## Publisher’s Note

All claims expressed in this article are solely those of the authors and do not necessarily represent those of their affiliated organizations, or those of the publisher, the editors and the reviewers. Any product that may be evaluated in this article, or claim that may be made by its manufacturer, is not guaranteed or endorsed by the publisher.
