# Supplementary material for: Clear Improvement in Real-World Chronic Myeloid Leukemia Survival: A Comparison With Randomized Controlled Trials
Source: Front Oncol. 2022 Jul 14;12:892684. doi: 10.3389/fonc.2022.892684 (PMC9333088; doi:10.3389/fonc.2022.892684)
Supplement: Supplementary file 1 [file DataSheet_1.docx]

**Supplementary Materials**

**Tables**

**Suppl Mat Table 1. Quality indicators and distribution of hematological malignancies for patients (15-64 years) diagnosed in 2000-2013, by Cancer Registry (CR). CRs with national coverage in bold. EUROCARE-6 study dataset.**

| **Area/Country** | | **Cancer registry (CR)** | **Overall period of diagnosis^1^** | **All haematological malignancies** | | | **Myeloid malignancies^3^ 2000-2013** | | | | | | **Exclusion criteria^5^** |
| --- | --- | --- | --- | --- | --- | --- | --- | --- | --- | --- | --- | --- | --- |
|  |  |  |  |  |  |  |  | | | **CML Cases included in survival analysis^4^** | | |  |
|  |  |  |  | **Eligible cases without major errors** | **% DCO and autopsy** | **% Lost to follow-up^2^** | **Cases 2000-2013** | **% MV** | **% NOS** | **CML total** | **CML NOS (9863) cases (%)** | **CML Ph+ (9875) cases (%)** |  |
| DENMARK | **Denmark** | 1978-2014 | 14,123 | 0.0 | 0.6 | 3,404 | 98.9 | 1.3 | 470 | 122 (26) | 348 (74) |  |  |
|  | FINLAND | **Finland** | 1978-2013 | 12,925 | 0.0 | 0.0 | 2,309 | 90.9 | 6.9 | 304 | 300 (99) | 4 (1) |  |
|  | ICELAND | **Iceland** | 1978-2014 | 587 | 0.7 | 0.0 | 78 | 98.7 | 1.3 | 23 | 22 (96) | 1 (4) |  |
|  | NORWAY | **Norway** | 1978-2016 | 11,948 | 0.2 | 0.0 | 2,557 | 98.9 | 1.8 | 312 | 283 (91) | 29 (9) |  |
| **UK and Ireland** | IRELAND | **Ireland** | 1994-2012 | 9,253 | 0.5 | 0.0 | 1,986 | 98.6 | 5.7 | 240 | 234 (98) | 6 (3) |  |
|  | UK-ENGLAND | **UK-England** | 1995-2013 | 104,476 | 0.7 | 0.0 | 15,100 | 91.1 | 5.1 | 3,548 | 3,449 (97) | 99 (3) |  |
|  | UK-NORTHERN IRELAND | **UK-Northern Ireland** | 1993-2013 | 4,365 | 0.5 | 0.0 | 1,381 | 50.7 | 1.4 | - | - | - | B |
|  | UK-SCOTLAND | **UK-Scotland** | 1978-2013 | 13,636 | 0.2 | 0.2 | 3,564 | 95.2 | 0.8 | 344 | 335 (97) | 9 (3) |  |
|  | UK-WALES | **UK-Wales** | 1991-2012 | 6,013 | 0.7 | 0.0 | 959 | 76.1 | 3.1 | 229 | 229 (100) | 0 (0) |  |
| **Central Europe** | AUSTRIA | **Austria** | 1983-2012 | 14,588 | 1.9 | 0.0 | 2,629 | 96.8 | 4.1 | 623 | 541 (87) | 82 (13) |  |
|  | BELGIUM | **Belgium** | 2004-2013 | 21,913 | 0.0 | 1.1 | 5,727 | 99.9 | 1.1 | 772 | 426 (55) | 346 (45) |  |
|  | FRANCE | Bas Rhin | 1990-2014 | 2,922 | 0.0 | 0.6 | 698 | 99.1 | 1.1 | 100 | 16 (16) | 84 (84) |  |
|  |  | Basse Normandie, HM | 2002-2010 | 3,471 | 0.0 | 0.0 | 994 | 93.1 | 1.5 | 113 | 5 (4) | 108 (96) |  |
|  |  | Calvados | 1990-2014 | 174 | 0.0 | - | 42 | 100.0 | 7.1 | 2 | 2 (100) | 0 (0) |  |
|  |  | Cote dOr, HM | 1990-2014 | 1,524 | 0.0 | 0.0 | 393 | 100.0 | 0.3 | 53 | 0 (0) | 53 (100) |  |
|  |  | Doubs | 1990-2014 | 1,727 | 0.0 | 0.0 | 436 | 100.0 | 0.7 | 58 | 2 (3) | 56 (97) |  |
|  |  | Gironde, HM | 2002-2014 | 3,631 | 0.0 | 0.9 | 884 | 100.0 | 0.2 | 132 | 3 (2) | 129 (98) |  |
|  |  | Haut-Rhin | 1990-2014 | 2,095 | 0.0 | 0.7 | 511 | 100.0 | 1.6 | 83 | 24 (29) | 59 (71) |  |
|  |  | Herault | 1995-2014 | 2,811 | 0.0 | 1.7 | 729 | 100.0 | 0.5 | 111 | 30 (27) | 81 (73) |  |
|  |  | Isere | 1990-2014 | 3,213 | 0.0 | 1.3 | 791 | 100.0 | 0.6 | 108 | 12 (11) | 96 (89) |  |
|  |  | Lille Area | 2008-2014 | 867 | 0.0 | 0.0 | 255 | 100.0 | 1.6 | - | - | - | A |
|  |  | Limousin | 2009-2014 | 401 | 0.0 | - | 117 | 100.0 | 0.0 | - | - | - | A |
|  |  | Loire-Atlantique/Vendée | 1991-2014 | 5,471 | 0.0 | 1.1 | 1,195 | 100.0 | 0.8 | 195 | 36 (18) | 159 (82) |  |
|  |  | Manche | 1994-2014 | 154 | 0.0 | - | 45 | 100.0 | 4.4 | 8 | 8 (100) | 0 (0) |  |
|  |  | Poitou-Charentes | 2008-2014 | 2,328 | 0.0 | 1.8 | 496 | 99.4 | 0.6 | - | - | - | A |
|  |  | Somme | 1990-2014 | 1,493 | 0.0 | 1.7 | 435 | 99.8 | 0.7 | 66 | 10 (15) | 56 (85) |  |
|  |  | Tarn | 1990-2014 | 986 | 0.0 | 0.0 | 264 | 100.0 | 0.4 | 41 | 7 (17) | 34 (83) |  |
|  | GERMANY | Bremen | 2000-2013 | 1,660 | 3.3 | 4.3 | 377 | 98.9 | 0.5 | 51 | 19 (37) | 32 (63) |  |
|  |  | Common Cancer Registry of 4 Federal States^6^ | 2002-2013 | 23,555 | 3.0 | 0.0 | 5,493 | 99.1 | 3.1 | 705 | 442 (63) | 263 (37) |  |
|  |  | Hamburg | 1998-2012 | 3,852 | 1.6 | 8.2 | 587 | 99.1 | 2.6 | 147 | 131 (89) | 16 (11) |  |
|  |  | Lower Saxony | 2005-2012 | 10,299 | 4.2 | 0.00* | 1,688 | 86.6 | 2.9 | - | - | - | A |
|  |  | Rhineland-Palatinate | 2004-2012 | 5,149 | 5.8 | 0.00* | 1,198 | 93.2 | 2.1 | 198 | 188 (95) | 10 (5) |  |
|  |  | Saarland | 1993-2012 | 2,342 | 1.8 | 0.00* | 521 | 99.6 | 1.7 | 77 | 77 (100) | 0 (0) |  |
|  |  | Schleswig-Holstein | 2003-2012 | 5,403 | 4.2 | 0.00* | 1,062 | 94.5 | 1.2 | 158 | 117 (74) | 41 (26) |  |
|  | SWITZERLAND | Friburg | 2006-2013 | 411 | 0.0 | 0.0 | 87 | 100.0 | 3.4 | - | - | - | A |
|  |  | Geneva | 1978-2013 | 1,195 | 0.1 | 14.6 | 257 | 100.0 | 1.9 | - | - | - | C |
|  |  | Graubunden and Glarus | 1989-2013 | 579 | 0.2 | 6.5 | 115 | 100.0 | 2.6 | 19 | 17 (89) | 2 (11) |  |
|  |  | Eastern Switzerland | 1981-2013 | 1,262 | 0.5 | 3.8 | 236 | 100.0 | 2.1 | 51 | 45 (88) | 6 (12) |  |
|  |  | Ticino | 2000-2012 | 901 | 0.1 | 5.6 | 219 | 100.0 | 1.8 | 33 | 15 (45) | 18 (55) |  |
|  | THE NETHERLANDS | **The Netherlands** | 1989-2013 | 41,644 | 0.1 | 0.0 | 9,759 | 99.9 | 0.6 | 1,199 | 152 (13) | 1,047 (87) |  |
| **Southern Europe** | CROATIA | **Croatia** | 2000-2012 | 7,146 | 0.0 | 0.00* | 1,178 | 100.0 | 18.1 | 265 | 265 (100) | 0 (0) |  |
|  | CYPRUS | **Cyprus** | 2004-2014 | 1,428 | 2.7 | 0.0 | 232 | 100.0 | 3.0 | 38 | 36 (95) | 2 (5) |  |
|  | ITALY | Alto Adige | 1995-2010 | 869 | 0.2 | 0.0 | 193 | 100.0 | 3.1 | 17 | 0 (0) | 17 (100) |  |
|  |  | Barletta_Andria_Trani | 2006-2012 | 655 | 0.2 | 1.5 | 229 | 99.1 | 0.4 | - | - | - | A |
|  |  | Basilicata | 2006-2010 | 678 | 0.1 | 4.0 | 239 | 95.0 | 4.2 | - | - | - | A |
|  |  | Bergamo | 2007-2012 | 1,334 | 0.1 | 1.3 | 245 | 99.6 | 0.8 | - | - | - | A |
|  |  | Biella | 1995-2010 | 690 | 0.0 | 1.0 | 191 | 97.9 | 0.5 | 12 | 10 (83) | 2 (17) |  |
|  |  | Brescia | 1999-2010 | 2,118 | 0.0 | 1.9 | 290 | 94.1 | 9.3 | 65 | 65 (100) | 0 (0) |  |
|  |  | Catania-Messina-Enna | 2003-2013 | 4,455 | 0.1 | 3.4 | 1,259 | 99.5 | 4.7 | 152 | 126 (83) | 26 (17) |  |
|  |  | Catanzaro | 2003-2009 | 518 | 0.0 | 0.4 | 171 | 90.6 | 3.5 | 25 | 25 (100) | 0 (0) |  |
|  |  | Como | 2003-2011 | 1,067 | 0.1 | 1.0 | 238 | 97.1 | 2.1 | 31 | 31 (100) | 0 (0) |  |
|  |  | Cremona | 2005-2010 | 420 | 0.7 | 22.1* | 83 | 85.5 | 12.0 | - | - | - | A |
|  |  | Ferrara | 1991-2011 | 992 | 0.2 | 2.1 | 247 | 100.0 | 2.4 | 26 | 26 (100) | 0 (0) |  |
|  |  | Firenze-Prato | 1985-2010 | 2,944 | 0.0 | 2.4 | 808 | 66.3 | 9.2 | - | - | - | B |
|  |  | Friuli Venezia Giulia | 1995-2010 | 2,329 | 0.6 | 1.6 | 343 | 100.0 | 3.8 | 75 | 75 (100) | 0 (0) |  |
|  |  | Genova | 1986-2010 | 2,337 | 0.1 | 0.0 | 650 | 73.1 | 2.8 | 57 | 55 (96) | 2 (4) |  |
|  |  | Latina | 1996-2012 | 1,327 | 0.5 | 6.0 | 308 | 79.5 | 1.9 | 43 | 37 (86) | 6 (14) |  |
|  |  | Lodi | 2003-2010 | 915 | 0.3 | 1.7 | 129 | 99.2 | 5.4 | 29 | 28 (97) | 1 (3) |  |
|  |  | Mantova | 1999-2010 | 835 | 0.1 | 0.5 | 123 | 100.0 | 5.7 | 26 | 26 (100) | 0 (0) |  |
|  |  | Modena | 1988-2013 | 2,202 | 0.0 | 0.0 | 518 | 99.0 | 1.2 | 86 | 37 (43) | 49 (57) |  |
|  |  | Monza and Brianza | 2007-2012 | 1,079 | 0.3 | 0.8 | 291 | 94.5 | 4.1 | - | - | - | A |
|  |  | Napoli | 1996-2013 | 2,362 | 0.2 | 1.0 | 652 | 95.7 | 7.7 | 75 | 49 (65) | 26 (35) |  |
|  |  | Nuoro | 2003-2012 | 527 | 0.0 | 0.0 | 114 | 100.0 | 0.0 | 14 | 14 (100) | 0 (0) |  |
|  |  | Palermo | 2003-2013 | 2,677 | 0.2 | 0.2 | 712 | 95.2 | 7.0 | 95 | 94 (99) | 1 (1) |  |
|  |  | Parma | 1978-2014 | 1,427 | 0.0 | 3.1 | 314 | 100.0 | 0.6 | 44 | 26 (59) | 18 (41) |  |
|  |  | Piacenza | 2006-2014 | 614 | 0.2 | 3.3 | 175 | 97.1 | 1.7 | - | - | - | A |
|  |  | Ragusa | 1981-2012 | 1,139 | 0.0 | 0.7 | 375 | 99.7 | 4.3 | 45 | 44 (98) | 1 (2) |  |
|  |  | Reggio Emilia | 1996-2014 | 1,695 | 0.0 | 3.4 | 407 | 98.8 | 1.0 | 68 | 30 (44) | 38 (56) |  |
|  |  | Romagna | 1986-2014 | 3,717 | 0.5 | 0.0 | 934 | 99.0 | 3.5 | 96 | 87 (91) | 9 (9) |  |
|  |  | Salerno | 1996-2010 | 2,267 | 0.1 | 5.6 | 571 | 96.1 | 4.9 | 77 | 76 (99) | 1 (1) |  |
|  |  | Sassari | 1992-2011 | 1,123 | 0.0 | 0.0 | 209 | 98.6 | 1.4 | 42 | 42 (100) | 0 (0) |  |
|  |  | Siracusa | 1999-2012 | 897 | 0.0 | 2.1 | 222 | 90.5 | 13.5 | 27 | 25 (93) | 2 (7) |  |
|  |  | Sondrio | 1998-2013 | 577 | 0.0 | 0.7 | 156 | 84.0 | 4.5 | 20 | 20 (100) | 0 (0) |  |
|  |  | Taranto | 2006-2012 | 853 | 0.1 | 2.5 | 250 | 95.6 | 1.6 | - | - | - | A |
|  |  | Trapani | 2002-2010 | 654 | 0.2 | 0.5 | 164 | 100.0 | 2.4 | 33 | 29 (88) | 4 (12) |  |
|  |  | Trento | 1995-2010 | 876 | 0.1 | 0.6 | 165 | 97.6 | 9.1 | 39 | 39 (100) | 0 (0) |  |
|  |  | Umbria | 1994-2013 | 2,626 | 0.0 | 0.8 | 692 | 98.7 | 4.9 | 96 | 96 (100) | 0 (0) |  |
|  |  | Varese | 1978-2012 | 2,261 | 0.2 | 1.8 | 348 | 92.2 | 12.9 | 85 | 83 (98) | 2 (2) |  |
|  |  | Veneto | 1987-2010 | 5,616 | 0.4 | 1.2 | 1,244 | 96.1 | 2.7 | 147 | 145 (99) | 2 (1) |  |
|  |  | Viterbo | 2006-2010 | 362 | 0.3 | 1.1* | 106 | 87.7 | 5.7 | - | - | - | A |
|  | MALTA | **Malta** | 1993-2013 | 955 | 0.8 | 0.0 | 192 | 99.0 | 7.8 | 19 | 19 (100) | 0 (0) |  |
|  | PORTUGAL | Central Portugal | 2007-2010 | 1,100 | 0.0 | 0.0 | 196 | 73.5 | 5.6 | - | - | - | A |
|  |  | Northern Portugal | 2000-2010 | 4,746 | 0.0 | 0.1 | 939 | 99.9 | 3.8 | 145 | 124 (86) | 21 (14) |  |
|  |  | Southern Portugal | 2000-2012 | 10,328 | 0.0 | 1.7 | 2,055 | 99.9 | 7.8 | 305 | 262 (86) | 43 (14) |  |
|  | SLOVENIA | **Slovenia** | 1983-2012 | 4,001 | 0.5 | 0.0 | 1,000 | 100.0 | 1.6 | 102 | 93 (91) | 9 (9) |  |
|  | SPAIN | Balearic Islands | 1988-2012 | 1,713 | 0.3 | 1.3 | 456 | 99.8 | 1.3 | 65 | 41 (63) | 24 (37) |  |
|  |  | Basque Country | 1986-2012 | 5,104 | 0.2 | 0.0 | 1,163 | 99.1 | 6.0 | 174 | 131 (75) | 43 (25) |  |
|  |  | Canarie | 1996-2011 | 3,381 | 0.6 | 0.0 | 645 | 99.7 | 1.6 | 97 | 87 (90) | 10 (10) |  |
|  |  | Castellon | 2004-2012 | 737 | 0.1 | 0.0 | 199 | 100.0 | 4.0 | 30 | 29 (97) | 1 (3) |  |
|  |  | Girona | 1994-2014 | 1,796 | 0.6 | 2.4 | 475 | 99.8 | 0.4 | 64 | 14 (22) | 50 (78) |  |
|  |  | Granada | 1985-2012 | 1,596 | 0.2 | 0.0 | 363 | 100.0 | 2.8 | 51 | 27 (53) | 24 (47) |  |
|  |  | Murcia | 1990-2010 | 2,170 | 0.4 | 0.5 | 492 | 98.8 | 4.3 | 90 | 90 (100) | 0 (0) |  |
|  |  | Navarra | 1978-2010 | 1,027 | 0.6 | 0.3 | 189 | 98.4 | 2.1 | 22 | 21 (95) | 1 (5) |  |
|  |  | Tarragona | 1982-2011 | 1,371 | 1.3 | 0.0 | 336 | 100.0 | 3.0 | 53 | 35 (66) | 18 (34) |  |
| **Eastern Europe** | BULGARIA | **Bulgaria** | 1993-2013 | 11,118 | 5.5 | 0.0 | 2,899 | 100.0 | 8.2 | 690 | 690 (100) | 0 (0) |  |
|  | CZECH REPUBLIC | **Czech Republic** | 1994-2013 | 19,870 | 3.7 | 0.0 | 2,975 | 72.2 | 25.8 | 586 | 468 (80) | 118 (20) |  |
|  | ESTONIA | **Estonia** | 1978-2012 | 2,367 | 1.0 | 0.8 | 528 | 100.0 | 1.9 | 88 | 84 (95) | 4 (5) |  |
|  | LATVIA | **Latvia** | 2000-2013 | 3,221 | 0.0 | 0.0 | 695 | 99.9 | 11.4 | 146 | 146 (100) | 0 (0) |  |
|  | LITHUANIA | **Lithuania** | 1993-2012 | 6,353 | 1.1 | 1.8 | 2,012 | 99.3 | 3.6 | 325 | 250 (77) | 75 (23) |  |
|  | POLAND | **Poland** | 2001-2013 | 49,773 | 1.0 | 0.0 | 8,093 | 95.6 | 9.8 | 2,197 | 2,197 (100) | 0 (0) |  |
|  | SLOVAKIA | **Slovakia** | 1978-2010 | 8,460 | 3.6 | 0.0 | 2,067 | 100.0 | 2.0 | 311 | 257 (83) | 54 (17) |  |
| **Total 101 CRs** | | | | **555,142** | **1.0** | **2.6** | **113,322** | **95.2** | **4.5** | **-** | **-** | **-** |  |
|  |  | **Total 84 CRs eligible for survival analysis** |  | **525,237** | **1.0** | **1.7** | **106,419** | **96.1** | **4.5** | **18,083** | **14,105 (78)** | **3,978 (22)** |  |

Autopsy: cases incidentally detected at autopsy; CML: chronic myeloid leukemia; CR: cancer registry; DCO, death certificate only; HM, hematological malignancies; ICD-O-3: International Classification of Disease for Oncology, 3rd edition; MV: microscopically verified; NOS: not otherwise specified; Ph, Philadelphia chromosome.

^1^ CRs period of diagnosis refers to overall data sent by each Cancer Registry.

^2^ Proportion of patients diagnosed in 2005-08, censored at 31/12/2013 with less than five years of follow-up. The proportion is calculated for cases diagnosed in 2005-07 where follow up closing date was 31/12/2012 (*).

^3^ International Classification of Disease for Oncology, 3rd edition (ICD-O-3) codes for Myeloid malignancies: 9740-9742,9800-9801,9805-9809,9840,9860-9861,9863,9865-9867,9869-9876,9891,9895-9898,9910-9911,9920,9930-9931,9945-9946,9950,9960-9964,9966,9975,9980,9982-9987,9989,9991-9992.

^4^ ICD-O-3 codes of CML cases eligible for the survival analysis: 9863 (CML with no cytogenetic information, CML NOS), 9875 (BCR/ABL1-positive CML).

^5^ Exclusion criteria: A = partial or missing time coverage for 2000-2006; B = Quality indicators issues (<70% MV); C = Quality indicators issues (>10% censored).

^6^ Four Federal States: Brandenburg, Mecklenburg-Western Pomerania and the Free States of Saxony and Thuringia.

The table gives a global evaluation of quality and completeness of collected data.

**Suppl Mat Table 2. 5-year crude overall survival of CML cases (15-64 years) diagnosed in 2000-2006 and 2007-2013 by European region, country, morphology code and comparisons. EUROCARE-6 study dataset.**

| **Country/Area** | **ICD-O-3 CML code^1^** | **2000-2006** | | | | | **2007-2013** | | | | | **Absolute difference 2000-2006 vs 2007-2013** | **p-value** | **Absolute difference 2000-2006 CML 9875 vs 9863** | **p-value** | **Absolute difference 2007-2013 CML 9875 vs 9863** | **p-value** |
| --- | --- | --- | --- | --- | --- | --- | --- | --- | --- | --- | --- | --- | --- | --- | --- | --- | --- |
|  |  | **N at start** | **N_5_** | **OS** | **95%CI** | | **N at start** | **N_5_** | **OS** | **95%CI** | |  |  |  |  |  |  |
| **Northern Europe (4 CRs)** | **CML (9863, 9875)** | **534** | **438** | **80.5** | **77.2** | **83.9** | **575** | **314** | **89.2** | **86.4** | **92.2** | **8.8**** | **<0.001** |  |  |  |  |
|  | CML Nos (9863) | 429 | 342 | 78.5 | 74.7 | 82.5 | 298 | 166 | 89.0 | 85.3 | 92.9 | **10.5**** | **<0.001** |  |  |  |  |
|  | CML positive (9875) | 105 | 96 | 88.6 | 82.7 | 94.9 | 277 | 148 | 89.3 | 85.0 | 93.9 | 0.8 | 0.843 | **10.1**** | **0.006** | 0.3 | 0.908 |
| Denmark | CML (9863, 9875) | 225 | 186 | 80.8 | 75.8 | 86.1 | 245 | 135 | 88.7 | 84.0 | 93.6 | **7.9*** | **0.028** |  |  |  |  |
|  | CML Nos (9863) | 121 | 91 | 74.1 | 66.6 | 82.4 | 1 | - | - | - | - | - | - |  |  |  |  |
|  | CML positive (9875) | 104 | 95 | 88.5 | 82.5 | 94.8 | 244 | 135 | 88.7 | 84.0 | 93.6 | 0.2 | 0.956 |  |  |  |  |
| Finland | CML (9863, 9875) | 165 | 135 | 80.0 | 74.1 | 86.3 | 139 | 73 | 86.0 | 79.8 | 92.6 | 6.0 | 0.187 |  |  |  |  |
|  | CML Nos (9863) | 165 | 135 | 80.0 | 74.1 | 86.3 | 135 | 70 | 85.5 | 79.2 | 92.4 | 5.5 | 0.228 |  |  |  |  |
|  | CML positive (9875) | - | - | - | - | - | 4 | 3 | - | - | - | - | - |  |  |  |  |
| Iceland | CML (9863, 9875) | 11 | 9 | - | - | - | 12 | 6 | - | - | - | - | - |  |  |  |  |
|  | CML Nos (9863) | 11 | 9 | - | - | - | 11 | 6 | - | - | - | - | - |  |  |  |  |
|  | CML positive (9875) | - | - | - | - | - | 1 | - | - | - | - | - | - |  |  |  |  |
| Norway | CML (9863, 9875) | 133 | 108 | 80.5 | 74.0 | 87.5 | 179 | 100 | 91.8 | 87.7 | 96.0 | **11.3**** | **0.005** |  |  |  |  |
|  | CML Nos (9863) | 132 | 107 | 80.3 | 73.8 | 87.4 | 151 | 90 | 91.1 | 86.6 | 95.8 | **10.8*** | **0.010** |  |  |  |  |
|  | CML positive (9875) | 1 | 1 | - | - | - | 28 | 10 | - | - | - | - | - |  |  |  |  |
| **UK and Ireland (4 CRs)** | **CML (9863, 9875)** | **2,001** | **1,488** | **72.2** | **70.3** | **74.2** | **2,360** | **1,187** | **86.9** | **85.3** | **88.4** | **14.7**** | **<0.001** |  |  |  |  |
|  | CML Nos (9863) | 1,918 | 1,414 | 71.6 | 69.6 | 73.6 | 2,329 | 1,169 | 86.7 | 85.2 | 88.3 | **15.1**** | **<0.001** |  |  |  |  |
|  | CML positive (9875) | 83 | 74 | 86.7 | 79.8 | 94.4 | 31 | 18 | 96.8 | 90.8 | 103.2 | **10.0*** | **0.040** | **15.2**** | **<0.001** | **10.0**** | **0.002** |
| Ireland | CML (9863, 9875) | 117 | 97 | 79.5 | 72.5 | 87.2 | 123 | 58 | 90.7 | 85.3 | 96.5 | **11.2*** | **0.017** |  |  |  |  |
|  | CML Nos (9863) | 117 | 97 | 79.5 | 72.5 | 87.2 | 117 | 57 | 90.3 | 84.7 | 96.3 | **10.9*** | **0.023** |  |  |  |  |
|  | CML positive (9875) | - | - | - | - | - | 6 | 1 | - | - | - | - | - |  |  |  |  |
| UK-England | CML (9863, 9875) | 1,596 | 1,167 | 70.9 | 68.7 | 73.2 | 1,952 | 982 | 86.5 | 84.8 | 88.2 | **15.5**** | **<0.001** |  |  |  |  |
|  | CML Nos (9863) | 1,515 | 1,095 | 70.1 | 67.8 | 72.4 | 1,934 | 967 | 86.3 | 84.6 | 88.0 | **16.2**** | **<0.001** |  |  |  |  |
|  | CML positive (9875) | 81 | 72 | 86.4 | 79.3 | 94.2 | 18 | 15 | - | - | - | - | - |  |  |  |  |
| UK-Scotland | CML (9863, 9875) | 166 | 139 | 83.1 | 77.6 | 89.0 | 178 | 87 | 87.8 | 82.1 | 93.7 | 4.6 | 0.263 |  |  |  |  |
|  | CML Nos (9863) | 164 | 137 | 82.9 | 77.3 | 88.9 | 171 | 85 | 87.9 | 82.3 | 94.0 | 5.0 | 0.230 |  |  |  |  |
|  | CML positive (9875) | 2 | 2 | - | - | - | 7 | 2 | - | - | - | - | - |  |  |  |  |
| UK-Wales | CML (9863, 9875) | 122 | 85 | 67.2 | 59.4 | 76.1 | 107 | 60 | 88.2 | 81.7 | 95.2 | **21.0**** | **<0.001** |  |  |  |  |
|  | CML Nos (9863) | 122 | 85 | 67.2 | 59.4 | 76.1 | 107 | 60 | 88.2 | 81.7 | 95.2 | **21.0**** | **<0.001** |  |  |  |  |
|  | CML positive (9875) | - | - | - | - | - | - | - | - | - | - | - | - |  |  |  |  |
| **Central Europe (25 CRs)** | **CML (9863, 9875)** | **2,186** | **1,829** | **82.6** | **81.0** | **84.2** | **2,917** | **1,407** | **88.5** | **87.1** | **89.9** | **5.9**** | **<0.001** |  |  |  |  |
|  | CML Nos (9863) | 1,174 | 948 | 79.5 | 77.3 | 81.9 | 1,151 | 537 | 85.6 | 83.3 | 88.0 | **6.1**** | **<0.001** |  |  |  |  |
|  | CML positive (9875) | 1,012 | 881 | 86.0 | 83.9 | 88.2 | 1,766 | 870 | 90.3 | 88.7 | 92.0 | **4.3**** | **0.002** | **6.5**** | **<0.001** | **4.7**** | **0.001** |
| Austria | CML (9863, 9875) | 347 | 262 | 74.6 | 70.2 | 79.4 | 276 | 146 | 84.2 | 79.6 | 89.1 | **9.5**** | **0.005** |  |  |  |  |
|  | CML Nos (9863) | 326 | 245 | 74.2 | 69.6 | 79.1 | 215 | 120 | 83.1 | 77.8 | 88.8 | **8.9*** | **0.016** |  |  |  |  |
|  | CML positive (9875) | 21 | 17 | - | - | - | 61 | 26 | 88.4 | 79.8 | 97.9 | - | - |  |  |  |  |
| Belgium | CML (9863, 9875) | 201 | 176 | 87.0 | 82.5 | 91.8 | 571 | 282 | 92.0 | 89.5 | 94.6 | 5.0 | 0.066 |  |  |  |  |
|  | CML Nos (9863) | 119 | 104 | 86.6 | 80.6 | 92.9 | 307 | 149 | 89.7 | 85.7 | 93.8 | 3.1 | 0.405 |  |  |  |  |
|  | CML positive (9875) | 82 | 72 | 87.7 | 80.9 | 95.1 | 264 | 133 | 94.7 | 91.8 | 97.6 | 6.9 | 0.077 |  |  |  |  |
| France (13 CRs Pool) | CML (9863, 9875) | 444 | 394 | 88.5 | 85.6 | 91.5 | 626 | 321 | 92.1 | 89.5 | 94.7 | 3.6 | 0.076 |  |  |  |  |
|  | CML Nos (9863) | 132 | 111 | 84.1 | 78.1 | 90.6 | 23 | 16 | - | - | - | - | - |  |  |  |  |
|  | CML positive (9875) | 312 | 283 | 90.3 | 87.1 | 93.7 | 603 | 305 | 92.3 | 89.7 | 95.0 | 2.0 | 0.361 |  |  |  |  |
| Germany (6 CRs Pool) | CML (9863, 9875) | 597 | 502 | 82.6 | 79.6 | 85.7 | 739 | 312 | 85.7 | 82.7 | 88.8 | 3.2 | 0.150 |  |  |  |  |
|  | CML Nos (9863) | 475 | 395 | 81.7 | 78.2 | 85.2 | 499 | 207 | 83.9 | 80.0 | 87.9 | 2.2 | 0.407 |  |  |  |  |
|  | CML positive (9875) | 122 | 107 | 86.1 | 80.1 | 92.4 | 240 | 105 | 89.5 | 84.9 | 94.3 | 3.4 | 0.384 |  |  |  |  |
| Switzerland (3CRs Pool) | CML (9863, 9875) | 51 | 47 | 90.2 | 82.4 | 98.7 | 52 | 26 | 85.8 | 74.4 | 99.0 | -4.4 | 0.561 |  |  |  |  |
|  | CML Nos (9863) | 40 | 36 | 87.5 | 77.8 | 98.4 | 37 | 16 | 90.2 | 80.1 | 101.5 | 2.7 | 0.722 |  |  |  |  |
|  | CML positive (9875) | 11 | 11 | - | - | - | 15 | 10 | - | - | - | - | - |  |  |  |  |
| The Netherlands | CML (9863, 9875) | 546 | 448 | 80.4 | 77.1 | 83.8 | 653 | 320 | 87.2 | 84.2 | 90.2 | **6.7**** | **0.003** |  |  |  |  |
|  | CML Nos (9863) | 82 | 57 | 67.1 | 57.6 | 78.1 | 70 | 29 | 86.5 | 78.7 | 95.1 | **19.5**** | **0.003** |  |  |  |  |
|  | CML positive (9875) | 464 | 391 | 82.8 | 79.4 | 86.3 | 583 | 291 | 87.3 | 84.2 | 90.5 | 4.5 | 0.057 |  |  |  |  |
| **Southern Europe (44 CRs)** | **CML (9863, 9875)** | **1,738** | **1,396** | **78.1** | **76.2** | **80.1** | **1,429** | **816** | **86.9** | **85.0** | **88.8** | **8.8**** | **<0.001** |  |  |  |  |
|  | CML Nos (9863) | 1,556 | 1,231 | 76.8 | 74.7 | 78.9 | 1,158 | 678 | 86.5 | 84.4 | 88.6 | **9.7**** | **<0.001** |  |  |  |  |
|  | CML positive (9875) | 182 | 165 | 89.0 | 84.6 | 93.7 | 271 | 138 | 89.0 | 85.0 | 93.2 | 0.0 | 0.992 | **12.2**** | **<0.001** | 2.5 | 0.289 |
| Croatia | CML (9863, 9875) | 154 | 100 | 59.7 | 52.5 | 68.0 | 111 | 25 | 68.6 | 57.7 | 81.5 | 8.8 | 0.220 |  |  |  |  |
|  | CML Nos (9863) | 154 | 100 | 59.7 | 52.5 | 68.0 | 111 | 25 | 68.6 | 57.7 | 81.5 | 8.8 | 0.220 |  |  |  |  |
|  | CML positive (9875) | - | - | - | - | - | - | - | - | - | - | - | - |  |  |  |  |
| Cyprus | CML (9863, 9875) | 10 | 9 | - | - | - | 28 | 19 | - | - | - | - | - |  |  |  |  |
|  | CML Nos (9863) | 9 | 8 | - | - | - | 27 | 18 | - | - | - | - | - |  |  |  |  |
|  | CML positive (9875) | 1 | 1 | - | - | - | 1 | 1 | - | - | - | - | - |  |  |  |  |
| Italy (29 CRs Pool) | CML (9863, 9875) | 906 | 752 | 81.3 | 78.8 | 83.9 | 741 | 441 | 88.3 | 85.8 | 90.8 | **7.0**** | **<0.001** |  |  |  |  |
|  | CML Nos (9863) | 816 | 671 | 80.4 | 77.7 | 83.2 | 624 | 394 | 88.5 | 85.8 | 91.2 | **8.1**** | **<0.001** |  |  |  |  |
|  | CML positive (9875) | 90 | 81 | 88.9 | 82.6 | 95.6 | 117 | 47 | 86.7 | 79.3 | 94.7 | -2.2 | 0.663 |  |  |  |  |
| Malta | CML (9863, 9875) | 12 | 9 | - | - | - | 7 | 2 | - | - | - | - | - |  |  |  |  |
|  | CML Nos (9863) | 12 | 9 | - | - | - | 7 | 2 | - | - | - | - | - |  |  |  |  |
|  | CML positive (9875) | - | - | - | - | - | - | - | - | - | - | - | - |  |  |  |  |
| Portugal (2 CRs Pool) | CML (9863, 9875) | 254 | 191 | 74.0 | 68.8 | 79.6 | 196 | 121 | 82.9 | 77.5 | 88.6 | **8.9*** | **0.025** |  |  |  |  |
|  | CML Nos (9863) | 241 | 179 | 73.0 | 67.6 | 78.8 | 145 | 93 | 83.0 | 76.8 | 89.7 | **10.0*** | **0.021** |  |  |  |  |
|  | CML positive (9875) | 13 | 12 | - | - | - | 51 | 28 | 82.5 | 72.0 | 94.4 | - | - |  |  |  |  |
| Slovenia | CML (9863, 9875) | 54 | 35 | 59.3 | 47.5 | 73.9 | 48 | 30 | 91.7 | 84.2 | 99.8 | **32.4**** | **<0.001** |  |  |  |  |
|  | CML Nos (9863) | 48 | 32 | 60.4 | 48.1 | 76.0 | 45 | 29 | 93.3 | 86.3 | 100.9 | **32.9**** | **<0.001** |  |  |  |  |
|  | CML positive (9875) | 6 | 3 | - | - | - | 3 | 1 | - | - | - | - | - |  |  |  |  |
| Spain (CRs Pool) | CML (9863, 9875) | 348 | 300 | 83.6 | 79.8 | 87.6 | 298 | 178 | 90.3 | 86.7 | 94.0 | **6.7*** | **0.014** |  |  |  |  |
|  | CML Nos (9863) | 276 | 232 | 81.5 | 77.1 | 86.2 | 199 | 117 | 88.1 | 83.4 | 93.1 | 6.6 | 0.053 |  |  |  |  |
|  | CML positive (9875) | 72 | 68 | 91.7 | 85.5 | 98.3 | 99 | 61 | 94.8 | 90.5 | 99.4 | 3.1 | 0.431 |  |  |  |  |
| **Eastern Europe (7 CRs)** | **CML (9863, 9875)** | **2,334** | **1,351** | **55.3** | **53.3** | **57.3** | **2,009** | **754** | **72.8** | **70.6** | **75.1** | **17.6**** | **<0.001** |  |  |  |  |
|  | CML Nos (9863) | 2,274 | 1,310 | 55.0 | 53.0 | 57.1 | 1,818 | 665 | 71.6 | 69.2 | 74.0 | **16.6**** | **<0.001** |  |  |  |  |
|  | CML positive (9875) | 60 | 41 | 66.7 | 55.7 | 79.7 | 191 | 89 | 84.7 | 78.6 | 91.2 | **18.0**** | **0.009** | 11.7 | 0.058 | **13.1**** | **<0.001** |
| Bulgaria | CML (9863, 9875) | 390 | 174 | 41.3 | 36.7 | 46.5 | 300 | 106 | 63.6 | 58.0 | 69.7 | **22.3**** | **<0.001** |  |  |  |  |
|  | CML Nos (9863) | 390 | 174 | 41.3 | 36.7 | 46.5 | 300 | 106 | 63.6 | 58.0 | 69.7 | **22.3**** | **<0.001** |  |  |  |  |
|  | CML positive (9875) | - | - | - | - | - | - | - | - | - | - | - | - |  |  |  |  |
| Czech Republic | CML (9863, 9875) | 336 | 228 | 66.1 | 61.2 | 71.3 | 250 | 81 | 75.0 | 68.5 | 82.2 | **9.0*** | **0.039** |  |  |  |  |
|  | CML Nos (9863) | 312 | 210 | 65.7 | 60.6 | 71.2 | 156 | 48 | 68.9 | 60.4 | 78.6 | 3.2 | 0.550 |  |  |  |  |
|  | CML positive (9875) | 24 | 18 | - | - | - | 94 | 33 | 86.0 | 77.2 | 95.8 | - | - |  |  |  |  |
| Estonia | CML (9863, 9875) | 53 | 30 | 54.7 | 42.8 | 69.9 | 35 | 19 | 69.0 | 54.5 | 87.3 | 14.2 | 0.186 |  |  |  |  |
|  | CML Nos (9863) | 53 | 30 | 54.7 | 42.8 | 69.9 | 31 | 19 | 69.6 | 54.8 | 88.5 | 14.9 | 0.173 |  |  |  |  |
|  | CML positive (9875) | - | - | - | - | - | 4 | - | - | - | - | - | - |  |  |  |  |
| Latvia | CML (9863, 9875) | 67 | 40 | 56.7 | 46.0 | 69.9 | 79 | 28 | 63.8 | 52.8 | 77.1 | 7.1 | 0.414 |  |  |  |  |
|  | CML Nos (9863) | 67 | 40 | 56.7 | 46.0 | 69.9 | 79 | 28 | 63.8 | 52.8 | 77.1 | 7.1 | 0.414 |  |  |  |  |
|  | CML positive (9875) | - | - | - | - | - | - | - | - | - | - | - | - |  |  |  |  |
| Lithuania | CML (9863, 9875) | 179 | 92 | 49.1 | 42.3 | 57.0 | 146 | 73 | 78.7 | 71.9 | 86.1 | **29.5**** | **<0.001** |  |  |  |  |
|  | CML Nos (9863) | 143 | 69 | 45.4 | 38.0 | 54.4 | 107 | 51 | 77.5 | 69.7 | 86.1 | **32.1**** | **<0.001** |  |  |  |  |
|  | CML positive (9875) | 36 | 23 | 63.9 | 50.0 | 81.7 | 39 | 22 | 83.0 | 71.2 | 96.8 | 19.1 | 0.064 |  |  |  |  |
| Poland | CML (9863, 9875) | 1,105 | 669 | 57.8 | 55.0 | 60.8 | 1,092 | 384 | 74.3 | 71.2 | 77.6 | **16.5**** | **<0.001** |  |  |  |  |
|  | CML Nos (9863) | 1,105 | 669 | 57.8 | 55.0 | 60.8 | 1,092 | 384 | 74.3 | 71.2 | 77.6 | **16.5**** | **<0.001** |  |  |  |  |
|  | CML positive (9875) | - | - | - | - | - | - | - | - | - | - | - | - |  |  |  |  |
| Slovakia | CML (9863, 9875) | 204 | 118 | 55.4 | 49.0 | 62.7 | 107 | 63 | 78.2 | 70.3 | 86.9 | **22.8**** | **<0.001** |  |  |  |  |
|  | CML Nos (9863) | 204 | 118 | 55.4 | 49.0 | 62.7 | 53 | 29 | 69.6 | 58.2 | 83.2 | 14.2 | 0.050 |  |  |  |  |
|  | CML positive (9875) | - | - | - | - | - | 54 | 34 | 86.0 | 75.7 | 97.7 | - | - |  |  |  |  |
| **European Pool (84 CRs)** | **CML (9863, 9875)** | **8,793** | **6,502** | **71.9** | **71.0** | **72.9** | **9,290** | **4,478** | **84.7** | **83.9** | **85.5** | **12.7**** | **<0.001** |  |  |  |  |
|  | CML Nos (9863) | 7,351 | 5,245 | 69.2 | 68.2 | 70.3 | 6,754 | 3,215 | 82.8 | 81.8 | 83.8 | **13.5**** | **<0.001** |  |  |  |  |
|  | CML positive (9875) | 1,442 | 1,257 | 85.8 | 84.1 | 87.7 | 2,536 | 1,263 | 89.8 | 88.4 | 91.2 | **3.9**** | **0.001** | **16.6**** | **<0.001** | **7.0**** | **<0.001** |

CI, confidence interval; CML: chronic myeloid leukemia; CR: cancer registrie; ICD-O-3: International Classification of Disease for Oncology, 3rd edition; N at start: number of CML cases alive at the beginning of the period; N_5_: number of CML cases alive at 5 years from diagnosis; OS: overall survival.

^1^ ICD-O-3 codes of CML cases eligible for the survival analysis: 9863 (CML with no cytogenetic information, CML NOS), 9875 (Ph+, *BCR/ABL1*-positive CML).

Survival estimates are not provided for strata including fewer than 10 cases.

** p-value <0.01, * p-value <0.05

**Suppl Mat Table 3. 5-year crude relative survival of CML cases (15-64 years) diagnosed in 2000-2006 and 2007-2013 by European region, country, morphology code and comparisons. EUROCARE-6 study dataset.**

| **Country/Area** | **ICD-O-3 CML code^1^** | **2000-2006** | | | | | **2007-2013** | | | | | **Absolute difference** | **p-value** |
| --- | --- | --- | --- | --- | --- | --- | --- | --- | --- | --- | --- | --- | --- |
|  |  | **N at start** | **N_5_** | **RS** | **95%CI** | | **N at start** | **N_5_** | **RS** | **95%CI** | |  |  |
| **Northern Europe (4 CRs)** | **CML (9863, 9875)** | **534** | **438** | **82.5** | **79.1** | **86.0** | **575** | **314** | **91.3** | **88.4** | **94.3** | **8.9**** | **<0.001** |
|  | CML Nos (9863) | 429 | 342 | 80.3 | 76.5 | 84.4 | 298 | 166 | 90.9 | 87.2 | 94.9 | **10.6**** | **<0.001** |
|  | CML positive (9875) | 105 | 96 | 91.1 | 85.1 | 97.6 | 277 | 148 | 91.6 | 87.1 | 96.3 | 0.5 | 0.907 |
| **DK_Denmark** | CML (9863, 9875) | 225 | 186 | 83.1 | 78.0 | 88.6 | 245 | 135 | 91.0 | 86.2 | 96.1 | **7.9*** | **0.033** |
|  | CML Nos (9863) | 121 | 91 | 76.2 | 68.6 | 84.8 | 1 | - | - | - | - | - | - |
|  | CML positive (9875) | 104 | 95 | 91.0 | 84.9 | 97.6 | 244 | 135 | 91.0 | 86.2 | 96.1 | 0.0 | 0.993 |
| **FI_Finland** | CML (9863, 9875) | 165 | 135 | 81.9 | 75.9 | 88.4 | 139 | 73 | 88.2 | 81.9 | 95.0 | 6.2 | 0.177 |
|  | CML Nos (9863) | 165 | 135 | 81.9 | 75.9 | 88.4 | 135 | 70 | 87.8 | 81.2 | 94.8 | 5.8 | 0.216 |
|  | CML positive (9875) | - | - | - | - | - | 4 | 3 | - | - | - | - | - |
| **IC_Iceland** | CML (9863, 9875) | 11 | 9 | - | - | - | 12 | 6 | - | - | - | - | - |
|  | CML Nos (9863) | 11 | 9 | - | - | - | 11 | 6 | - | - | - | - | - |
|  | CML positive (9875) | - | - | - | - | - | 1 | - | - | - | - | - | - |
| **NO_Norway** | CML (9863, 9875) | 133 | 108 | 81.9 | 75.3 | 89.1 | 179 | 100 | 93.5 | 89.3 | 97.8 | **11.5**** | **0.005** |
|  | CML Nos (9863) | 132 | 107 | 81.8 | 75.2 | 89.0 | 151 | 90 | 92.8 | 88.2 | 97.7 | **11.0**** | **0.010** |
|  | CML positive (9875) | 1 | 1 | 100.5 | 100.5 | 100.5 | 28 | 10 | 96.6 | 87.1 | 107.1 | 3.9 | 0.441 |
| **UK and Ireland (4 CRs)** | **CML (9863, 9875)** | **2,001** | **1,488** | **73.9** | **72.0** | **76.0** | **2,360** | **1,187** | **88.7** | **87.1** | **90.3** | **14.7**** | **<0.001** |
|  | CML Nos (9863) | 1,918 | 1,414 | 73.3 | 71.3 | 75.4 | 2,329 | 1,169 | 88.5 | 87.0 | 90.1 | **15.2**** | **<0.001** |
|  | CML positive (9875) | 83 | 74 | 88.5 | 81.4 | 96.3 | 31 | 18 | 99.1 | 92.9 | 105.6 | **10.5*** | **0.035** |
| **IR_Ireland** | CML (9863, 9875) | 117 | 97 | 81.6 | 74.4 | 89.5 | 123 | 58 | 92.7 | 87.2 | 98.6 | **11.1*** | **0.021** |
|  | CML Nos (9863) | 117 | 97 | 81.6 | 74.4 | 89.5 | 117 | 57 | 92.3 | 86.6 | 98.4 | **10.7*** | **0.028** |
|  | CML positive (9875) | - | - | - | - | - | 6 | 1 | - | - | - | - | - |
| **UK_England** | CML (9863, 9875) | 1,596 | 1,167 | 72.5 | 70.3 | 74.8 | 1,952 | 982 | 88.2 | 86.5 | 90.0 | **15.7**** | **<0.001** |
|  | CML Nos (9863) | 1,515 | 1,095 | 71.7 | 69.4 | 74.1 | 1,934 | 967 | 88.1 | 86.3 | 89.8 | **16.4**** | **<0.001** |
|  | CML positive (9875) | 81 | 72 | 88.2 | 80.9 | 96.1 | 18 | 15 | - | - | - | - | - |
| **UK_Scotland** | CML (9863, 9875) | 166 | 139 | 85.9 | 80.2 | 92.0 | 178 | 87 | 90.1 | 84.4 | 96.3 | 4.3 | 0.319 |
|  | CML Nos (9863) | 164 | 137 | 85.7 | 79.9 | 91.8 | 171 | 85 | 90.3 | 84.5 | 96.5 | 4.6 | 0.282 |
|  | CML positive (9875) | 2 | 2 | 103.4 | 103.4 | 103.4 | 7 | 2 | 88.6 | 65.5 | 119.9 | 14.8 | 0.280 |
| **UK_Wales** | CML (9863, 9875) | 122 | 85 | 68.8 | 60.8 | 77.9 | 107 | 60 | 90.3 | 83.6 | 97.5 | **21.5**** | **<0.001** |
|  | CML Nos (9863) | 122 | 85 | 68.8 | 60.8 | 77.9 | 107 | 60 | 90.3 | 83.6 | 97.5 | **21.5**** | **<0.001** |
|  | CML positive (9875) | - | - | - | - | - | - | - | - | - | - | - | - |
| **Central Europe (25 CRs)** | **CML (9863, 9875)** | **2,186** | **1,829** | **84.6** | **83.0** | **86.2** | **2,917** | **1,407** | **90.6** | **89.2** | **92.0** | **6.0**** | **<0.001** |
|  | CML Nos (9863) | 1,174 | 948 | 81.7 | 79.3 | 84.1 | 1,151 | 537 | 87.8 | 85.4 | 90.2 | **6.1**** | **<0.001** |
|  | CML positive (9875) | 1,012 | 881 | 88.0 | 85.8 | 90.2 | 1,766 | 870 | 92.4 | 90.7 | 94.1 | **4.4**** | **0.002** |
| **AT_Austria** | CML (9863, 9875) | 347 | 262 | 76.5 | 72.0 | 81.4 | 276 | 146 | 86.3 | 81.6 | 91.4 | **9.8**** | **0.005** |
|  | CML Nos (9863) | 326 | 245 | 76.1 | 71.4 | 81.2 | 215 | 120 | 85.2 | 79.8 | 91.0 | **9.1*** | **0.017** |
|  | CML positive (9875) | 21 | 17 | - | - | - | 61 | 26 | 90.7 | 81.9 | 100.4 | - | - |
| **BE_Belgium** | CML (9863, 9875) | 201 | 176 | 89.2 | 84.6 | 94.1 | 571 | 282 | 94.3 | 91.7 | 96.9 | 5.0 | 0.069 |
|  | CML Nos (9863) | 119 | 104 | 88.9 | 82.8 | 95.4 | 307 | 149 | 92.1 | 88.0 | 96.3 | 3.2 | 0.402 |
|  | CML positive (9875) | 82 | 72 | 89.8 | 82.8 | 97.4 | 264 | 133 | 96.8 | 93.9 | 99.8 | 7.0 | 0.081 |
| **FR_France (13 CRs Pool)** | CML (9863, 9875) | 444 | 394 | 90.5 | 87.5 | 93.6 | 626 | 321 | 94.3 | 91.7 | 97.0 | 3.8 | 0.063 |
|  | CML Nos (9863) | 132 | 111 | 86.1 | 79.9 | 92.7 | 23 | 16 | - | - | - | - | - |
|  | CML positive (9875) | 312 | 283 | 92.4 | 89.1 | 95.8 | 603 | 305 | 94.6 | 91.9 | 97.3 | 2.2 | 0.316 |
| **GE_Germany (6 CRs Pool)** | CML (9863, 9875) | 597 | 502 | 85.0 | 81.9 | 88.1 | 739 | 312 | 87.9 | 84.8 | 91.1 | 2.9 | 0.192 |
|  | CML Nos (9863) | 475 | 395 | 84.0 | 80.5 | 87.7 | 499 | 207 | 86.0 | 82.1 | 90.1 | 2.0 | 0.458 |
|  | CML positive (9875) | 122 | 107 | 88.7 | 82.6 | 95.2 | 240 | 105 | 91.7 | 87.0 | 96.6 | 3.0 | 0.456 |
| **SW_Switzerland (3CRs Pool)** | CML (9863, 9875) | 51 | 47 | 91.9 | 83.9 | 100.6 | 52 | 26 | 87.3 | 75.6 | 100.7 | 4.6 | 0.545 |
|  | CML Nos (9863) | 40 | 36 | 89.0 | 79.1 | 100.0 | 37 | 16 | 92.0 | 81.7 | 103.5 | 3.0 | 0.697 |
|  | CML positive (9875) | 11 | 11 | - | - | - | 15 | 10 | - | - | - | - | - |
| **NL_The Netherlands** | CML (9863, 9875) | 546 | 448 | 82.1 | 78.8 | 85.6 | 653 | 320 | - | - | - | **6.7**** | **0.004** |
|  | CML Nos (9863) | 82 | 57 | 68.9 | 59.2 | 80.2 | 70 | 29 | - | - | - | **19.2**** | **0.005** |
|  | CML positive (9875) | 464 | 391 | 84.4 | 81.0 | 88.0 | 583 | 291 | - | - | - | 4.5 | 0.064 |
| **Southern Europe (44 CRs)** | **CML (9863, 9875)** | **1,738** | **1,396** | **79.8** | **77.8** | **81.8** | **1,429** | **816** | **-** | **-** | **-** | **8.9**** | **<0.001** |
|  | CML Nos (9863) | 1,556 | 1,231 | 78.5 | 76.4 | 80.7 | 1,158 | 678 | - | - | - | **9.8**** | **<0.001** |
|  | CML positive (9875) | 182 | 165 | 90.5 | 86.0 | 95.3 | 271 | 138 | - | - | - | 0.0 | 0.998 |
| **CY_Cyprus** | CML (9863, 9875) | 10 | 9 | - | - | - | 28 | 19 | - | - | - | - | - |
|  | CML Nos (9863) | 9 | 8 | - | - | - | 27 | 18 | - | - | - | - | - |
|  | CML positive (9875) | 1 | 1 | - | - | - | 1 | 1 | - | - | - | - | - |
| **CR_Croatia** | CML (9863, 9875) | 154 | 100 | 62.3 | 54.7 | 70.9 | 111 | 25 | 70.9 | 59.7 | 84.2 | 8.6 | 0.248 |
|  | CML Nos (9863) | 154 | 100 | 62.3 | 54.7 | 70.9 | 111 | 25 | 70.9 | 59.7 | 84.2 | 8.6 | 0.248 |
|  | CML positive (9875) | - | - | - | - | - | - | - | - | - | - | - | - |
| **IT_Italy (29 CRs Pool)** | CML (9863, 9875) | 906 | 752 | 82.9 | 80.3 | 85.5 | 741 | 441 | 90.0 | 87.4 | 92.6 | **7.1**** | **<0.001** |
|  | CML Nos (9863) | 816 | 671 | 82.1 | 79.3 | 84.9 | 624 | 394 | 90.2 | 87.6 | 93.0 | **8.2**** | **<0.001** |
|  | CML positive (9875) | 90 | 81 | 90.3 | 84.0 | 97.2 | 117 | 47 | 88.0 | 80.6 | 96.2 | 2.3 | 0.656 |
| **ML_Malta** | CML (9863, 9875) | 12 | 9 | 75.9 | 54.8 | 105.2 | 7 | 2 | 69.9 | 41.0 | 119.0 | 6.0 | 0.791 |
|  | CML Nos (9863) | 12 | 9 | 75.9 | 54.8 | 105.2 | 7 | 2 | 69.9 | 41.0 | 119.0 | 6.0 | 0.791 |
|  | CML positive (9875) | - | - | - | - | - | - | - | - | - | - | - | - |
| **PT_Portugal (2 CRs Pool)** | CML (9863, 9875) | 254 | 191 | 75.7 | 70.4 | 81.4 | 196 | 121 | 84.7 | 79.3 | 90.6 | **9.0*** | **0.025** |
|  | CML Nos (9863) | 241 | 179 | 74.7 | 69.2 | 80.7 | 145 | 93 | 84.9 | 78.6 | 91.7 | **10.2*** | **0.022** |
|  | CML positive (9875) | 13 | 12 | - | - | - | 51 | 28 | 84.2 | 73.6 | 96.5 | - | - |
| **SL_Slovenia** | CML (9863, 9875) | 54 | 35 | 60.8 | 48.7 | 75.9 | 48 | 30 | 94.6 | 86.9 | 103.0 | **33.8**** | **<0.001** |
|  | CML Nos (9863) | 48 | 32 | 62.1 | 49.4 | 78.0 | 45 | 29 | 96.4 | 89.2 | 104.3 | **34.4**** | **<0.001** |
|  | CML positive (9875) | 6 | 3 | - | - | - | 3 | 1 | - | - | - | - | - |
| **SP_Spain (CRs Pool)** | CML (9863, 9875) | 348 | 300 | 85.2 | 81.3 | 89.2 | 298 | 178 | 91.9 | 88.3 | 95.7 | **6.8*** | **0.014** |
|  | CML Nos (9863) | 276 | 232 | 83.0 | 78.5 | 87.8 | 199 | 117 | 89.8 | 84.9 | 94.9 | 6.7 | 0.053 |
|  | CML positive (9875) | 72 | 68 | 93.3 | 87.0 | 100.0 | 99 | 61 | 96.4 | 92.0 | 101.1 | 3.1 | 0.438 |
| **Eastern Europe (7 CRs)** | **CML (9863, 9875)** | **2,334** | **1,351** | **57.6** | **55.5** | **59.7** | **2,009** | **754** | **75.9** | **73.6** | **78.3** | **18.3**** | **<0.001** |
|  | CML Nos (9863) | 2,274 | 1,310 | 57.3 | 55.2 | 59.4 | 1,818 | 665 | 74.6 | 72.2 | 77.2 | **17.4**** | **<0.001** |
|  | CML positive (9875) | 60 | 41 | 69.4 | 58.0 | 83.0 | 191 | 89 | 87.7 | 81.4 | 94.5 | **18.3*** | **0.010** |
| **BG_Bulgaria** | CML (9863, 9875) | 390 | 174 | 43.5 | 38.7 | 49.0 | 300 | 106 | 66.8 | 60.9 | 73.2 | **23.3**** | **<0.001** |
|  | CML Nos (9863) | 390 | 174 | 43.5 | 38.7 | 49.0 | 300 | 106 | 66.8 | 60.9 | 73.2 | **23.3**** | **<0.001** |
|  | CML positive (9875) | - | - | - | - | - | - | - | - | - | - | - | - |
| **CZ_Czech Republic** | CML (9863, 9875) | 336 | 228 | 68.2 | 63.2 | 73.7 | 250 | 81 | 77.7 | 70.9 | 85.1 | **9.5*** | **0.035** |
|  | CML Nos (9863) | 312 | 210 | 67.8 | 62.6 | 73.5 | 156 | 48 | 71.5 | 62.7 | 81.6 | 3.7 | 0.503 |
|  | CML positive (9875) | 24 | 18 | - | - | - | 94 | 33 | 88.6 | 79.5 | 98.7 | - | - |
| **EE_Estonia** | CML (9863, 9875) | 53 | 30 | 58.4 | 45.7 | 74.6 | 35 | 19 | 71.5 | 56.4 | 90.5 | 13.1 | 0.246 |
|  | CML Nos (9863) | 53 | 30 | 58.4 | 45.7 | 74.6 | 31 | 19 | 72.3 | 56.9 | 91.9 | 13.9 | 0.226 |
|  | CML positive (9875) | - | - | - | - | - | 4 | - | - | - | - | - | - |
| **LV_Latvia** | CML (9863, 9875) | 67 | 40 | 59.7 | 48.4 | 73.6 | 79 | 28 | 67.7 | 56.1 | 81.8 | 8.0 | 0.378 |
|  | CML Nos (9863) | 67 | 40 | 59.7 | 48.4 | 73.6 | 79 | 28 | 67.7 | 56.1 | 81.8 | 8.0 | 0.378 |
|  | CML positive (9875) | - | - | - | - | - | - | - | - | - | - | - | - |
| **LT_Lithuania** | CML (9863, 9875) | 179 | 92 | 51.7 | 44.5 | 60.0 | 146 | 73 | 83.0 | 75.8 | 90.7 | **31.3**** | **<0.001** |
|  | CML Nos (9863) | 143 | 69 | 47.8 | 40.0 | 57.3 | 107 | 51 | 82.2 | 73.9 | 91.3 | **34.3**** | **<0.001** |
|  | CML positive (9875) | 36 | 23 | 66.8 | 52.2 | 85.3 | 39 | 22 | 86.2 | 73.9 | 100.5 | 19.4 | 0.071 |
| **PL_Poland** | CML (9863, 9875) | 1,105 | 669 | 60.1 | 57.1 | 63.2 | 1,092 | 384 | 77.2 | 74.0 | 80.6 | **17.2**** | **<0.001** |
|  | CML Nos (9863) | 1,105 | 669 | 60.1 | 57.1 | 63.2 | 1,092 | 384 | 77.2 | 74.0 | 80.6 | **17.2**** | **<0.001** |
|  | CML positive (9875) | - | - | - | - | - | - | - | - | - | - | - | - |
| **SK_Slovakia** | CML (9863, 9875) | 204 | 118 | 57.6 | 50.9 | 65.1 | 107 | 63 | 80.9 | 72.8 | 89.9 | **23.3**** | **<0.001** |
|  | CML Nos (9863) | 204 | 118 | 57.6 | 50.9 | 65.1 | 53 | 29 | 71.5 | 59.8 | 85.5 | 13.9 | 0.063 |
|  | CML positive (9875) | - | - | - | - | - | 54 | 34 | 89.6 | 78.9 | 101.8 | - | - |
| **European Pool (84 CRs)** | **CML (9863, 9875)** | **8,793** | **6,502** | **74.0** | **73.0** | **74.9** | **9,290** | **4,478** | **86.9** | **86.0** | **87.7** | **12.9**** | **<0.001** |
|  | CML Nos (9863) | 7,351 | 5,245 | 71.2 | 70.1 | 72.3 | 6,754 | 3,215 | 85.0 | 84.0 | 86.0 | **13.8**** | **<0.001** |
|  | CML positive (9875) | 1,442 | 1,257 | 87.8 | 86.0 | 89.7 | 2,536 | 1,263 | 91.8 | 90.4 | 93.3 | **4.0**** | **0.001** |

CI, confidence interval; CML: chronic myeloid leukemia; CR: cancer registry; N at start: number of CML cases alive at the beginning of the period; N_5_: number of CML cases alive at 5 years from diagnosis; ICD-O-3: International Classification of Disease for Oncology, 3rd edition; RS: relative survival.

^1^ ICD-O-3 codes of CML cases eligible for the survival analysis: 9863 (CML with no cytogenetic information, CML NOS), 9875 (Ph+, *BCR/ABL1*-positive CML).

Survival estimates are not provided for strata including fewer than 10 cases.

** p-value<0.01, * p-value <0.05

**Suppl Mat Table 4. Conditional crude relative survival of CML cases (15-64 years) (9863, 9875 ICD-O-3 codes)^1^ diagnosed in 2000-2006 and 2007-2013 by European region and country. EUROCARE-6 study dataset.**

| **Country/Area** | **2000-2006** | | | **2007-2013** | | | **Absolute difference** | **p-value** | **Absolute difference: CCRS *vs* CCOS in 2000-2006** | **Absolute difference: CCRS *vs* CCOS in 2007-2013** |
| --- | --- | --- | --- | --- | --- | --- | --- | --- | --- | --- |
|  | **5-/3-year** | **95%CI** | | **5-/3-year** | **95%CI** | |  |  |  |  |
| **Northern Europe (4 CRS)** | **96.8** | **94.9** | **98.7** | **97.4** | **95.0** | **99.9** | 0.7 | 0.679 | 1.0 | **1.0** |
| Denmark | 95.4 | 92.2 | 98.9 | 94.9 | 91.1 | 98.9 | -0.5 | 0.837 | 1.2 | **1.1** |
| Finland | 97.3 | 94.2 | 100.6 | 97.1 | 90.9 | 103.7 | -0.2 | 0.946 | 1.0 | **1.1** |
| Iceland | - | - | - | - | - | - | - | - | - | - |
| Norway | 98.0 | 95.0 | 101.2 | 100.9 | 97.8 | 104.0 | 2.8 | 0.204 | 0.8 | **0.9** |
| **UK and Ireland**  **(4 CRs)** | **93.8** | **92.5** | **95.1** | **98.1** | **97.1** | **99.1** | **4.3**** | **<0.001** | 0.9 | **0.9** |
| Ireland | 94.0 | 89.1 | 99.2 | 99.5 | 96.7 | 102.4 | 5.5 | 0.066 | 1.0 | **0.9** |
| UK-England | 93.5 | 92.0 | 94.9 | 98.2 | 97.2 | 99.2 | **4.8**** | **<0.001** | 0.9 | **0.9** |
| UK-Scotland | 95.9 | 92.2 | 99.7 | 96.3 | 91.4 | 101.4 | 0.4 | 0.899 | 1.4 | **1.1** |
| UK-Wales | 95.2 | 90.4 | 100.3 | 97.6 | 92.3 | 103.1 | 2.4 | 0.522 | 0.9 | **1.0** |
| **Central Europe (25 CRs)** | **97.3** | **96.4** | **98.2** | **97.0** | **95.8** | **98.2** | -0.3 | 0.707 | 1.0 | **1.0** |
| Austria | 97.3 | 95.1 | 99.6 | 94.9 | 90.2 | 99.8 | -2.4 | 0.369 | 1.0 | **1.0** |
| Belgium | 97.7 | 95.1 | 100.4 | 99.2 | - | - | - | - | 1.1 | **1.1** |
| France (13 CRs Pool) | 98.2 | 96.6 | 99.8 | 97.6 | 95.4 | 99.8 | -0.6 | 0.657 | 1.0 | **1.0** |
| Germany (6 CRs Pool) | 97.5 | 95.8 | 99.2 | 96.2 | 93.4 | 99.2 | -1.2 | 0.475 | 1.2 | **1.1** |
| Switzerland (3CRs Pool) | 96.6 | 91.1 | 102.5 | 92.6 | 81.3 | 105.4 | -4.1 | 0.550 | 0.8 | **0.7** |
| The Netherlands | 96.1 | 94.2 | 98.1 | 96.5 | 94.0 | 99.1 | 0.4 | 0.800 | 0.9 | **0.8** |
| **Southern Europe (44 CRs)** | **95.2** | **94.0** | **96.4** | **98.1** | **96.4** | **99.8** | **2.9**** | **0.007** | 0.9 | **0.9** |
| Croatia | 89.2 | 83.0 | 95.8 | 93.1 | 80.1 | 108.2 | 3.9 | 0.620 | 1.6 | **1.2** |
| Cyprus | 100.4 | 100.4 | 100.5 | 96.0 | 87.7 | 105.2 | -4.4 | 0.325 | - | - |
| Italy (29 CRs Pool) | 96.1 | 94.6 | 97.6 | 98.5 | 96.2 | 100.8 | 2.4 | 0.091 | 0.8 | **0.8** |
| Malta | 90.4 | 73.5 | 111.2 | 101.0 | 63.9 | 159.9 | 10.6 | 0.677 | - | - |
| Portugal (2 CRs Pool) | 94.4 | 91.0 | 97.9 | 97.0 | 91.9 | 102.4 | 2.6 | 0.421 | 0.9 | **0.9** |
| Slovenia | 87.4 | 77.0 | 99.3 | 101.4 | - | - | - | - | 1.0 | **1.4** |
| Spain (CRs Pool) | 96.5 | 94.2 | 98.8 | 98.2 | 95.1 | 101.3 | 1.7 | 0.392 | 0.7 | **0.8** |
| **Eastern Europe (7 CRs)** | **88.1** | **86.4** | **89.9** | **95.0** | **92.9** | **97.1** | **6.9**** | **<0.001** | 1.5 | **1.6** |
| Bulgaria | 79.9 | 74.3 | 85.9 | 97.1 | 93.6 | 100.8 | **17.3**** | **<0.001** | 1.7 | **2.0** |
| Czech Republic | 93.8 | 90.4 | 97.2 | 94.9 | 87.5 | 102.9 | 1.2 | 0.786 | 1.3 | **1.5** |
| Estonia | 82.7 | 70.4 | 97.0 | 87.7 | 73.9 | 104.0 | 5.0 | 0.625 | 2.1 | **1.3** |
| Latvia | 84.2 | 73.7 | 96.1 | 94.9 | 85.3 | 105.6 | 10.7 | 0.163 | 1.6 | **2.2** |
| Lithuania | 83.9 | 76.8 | 91.6 | 97.1 | 94.9 | 99.4 | **13.3**** | **0.001** | 1.6 | **2.1** |
| Poland | 89.4 | 87.1 | 91.9 | 94.1 | 91.2 | 97.1 | **4.7*** | **0.015** | 1.4 | **1.5** |
| Slovakia | 89.7 | 84.2 | 95.6 | 96.4 | 88.9 | 104.6 | 6.7 | 0.173 | 1.4 | **1.4** |
| **European Pool (84 CRs)** | **94.0** | **93.4** | **94.6** | **97.2** | **96.5** | **97.8** | **3.2**** | **<0.001** | 1.1 | **1.1** |

CCOS: conditional crude overall survival; CCRS: conditional crude relative survival; CI, confidence interval; CML: chronic myeloid leukemia; CR: cancer registry; ICD-O-3: International Classification of Disease for Oncology, 3rd edition.

^1^ ICD-O-3 codes of CML cases eligible for the survival analysis: 9863 (CML with no cytogenetic information, CML NOS), 9875 (Ph+, *BCR/ABL1*-positive CML).

The crude 5-/3-year conditional overall or relative survival is the probability of being alive after 5 years, conditional on surviving 3 years after diagnosis. Survival estimates are not provided for strata including fewer than 10 cases.

**Suppl Mat Table 5. Glivec (imatinib, Novartis) (Table 5-A) and Tasigna (nilotinib, Novartis) (Table 5-B) TKIs commercial availability in Europe.**

CML: chronic myeloid leukemia; EMA, European Medicines Agency; NA, not available.

Data provided by Novartis Farma in 2015 and authorized for publication by Novartis Farma in 2021.

**Table 5-A**

| **Brand** | **Country** | **Indication** | **Product is officially Reimbursed  ( yes/no )** | **Date** |
| --- | --- | --- | --- | --- |
| **Glivec** | Albania | CML | Yes | Sep-09 |
| **Glivec** | Austria | CML | 1st line: NO 2nd line: NO | - |
| **Glivec** | Belgium | CML | 1st line: YES 2nd line: NO | Jun-05 |
| **Glivec** | Bosnia | CML | Yes | Jun-05 |
| **Glivec** | Bulgaria | CML | 1st line: YES 2nd line: NO | - |
| **Glivec** | Croatia | CML | 1st line: YES 2nd line: YES | Jun-03 |
| **Glivec** | Cyprus | CML | 1st line: YES 2nd line: YES | Jun-01 |
| **Glivec** | Czech Republic | CML | Yes | - |
| **Glivec** | Denmark | CML | 1st line: YES 2nd line: YES | EMA |
| **Glivec** | Estonia | CML | No | Jun-06 |
| **Glivec** | Finland | CML | 1st line: YES 2nd line: yes | Jun-02 |
| **Glivec** | France | CML | 1st line: YES 2nd line: YES | Jun-02 |
| **Glivec** | Germany | CML | 1st line: YES 2nd line: YES | EMA |
| **Glivec** | Greece | CML | 1st line: YES 2nd line: YES | Jun-05 |
| **Glivec** | Hungary | CML | 1st line: YES 2nd line: YES | Jun-01 |
| **Glivec** | Iceland | CML | 1st line: YES 2nd line: YES | Since launch |
| **Glivec** | Ireland | CML | 1st line: YES 2nd line: YES | Since launch |
| **Glivec** | Israel | CML | 1st line:  2nd line: | Jun-99 |
| **Glivec** | Italy | CML | Yes | Jan-02 |
| **Glivec** | Kosovo | CML | Yes | Jul-05 |
| **Glivec** | Latvia | CML | 1st line: YES | - |
| **Glivec** | Lithuania | CML | 1st line: NO 2nd line: YES | Oct-12 |
| **Glivec** | Luxembourg | CML | 1st line: NA 2nd line: NA | - |
| **Glivec** | Macedonia | CML | Yes | Jun-10 |
| **Glivec** | Malta | CML | 1st line: YES 2nd line: YES | Jun-05 |
| **Glivec** | Montenegro | CML | Yes | Jul-05 |
| **Glivec** | Netherlands | CML | Yes | Jun-05 |
| **Glivec** | Norway | CML | 1st line: YES 2nd line: YES | EMA + 2 months |
| **Glivec** | Poland | CML | 1st line: YES 2nd line: NO | Jun-05 |
| **Glivec** | Portugal | CML | 1st line: YES 2nd line: YES | - |
| **Glivec** | Romania | CML | 1st line: YES 2nd line: YES | - |
| **Glivec** | Serbia | CML | No | Jun-05 |
| **Glivec** | Slovakia | CML | Yes | - |
| **Glivec** | Slovenia | CML | 1st line: YES 2nd line: YES | Jun-05 |
| **Glivec** | Spain | CML | 1st line: yes 2nd line:yes | Jun-05 |
| **Glivec** | Sweden | CML | 1st line: YES 2nd line: YES | - |
| **Glivec** | Switzerland | CML | 1st line: YES 2nd line: YES | - |
| **Glivec** | United Kingdom | CML | 1st line: YES 2nd line: YES | - |

**Table 5-B**

| **Tasigna** | Albania | 1st line | No | - |
| --- | --- | --- | --- | --- |
| **Tasigna** | Croatia | 1st line | No | - |
| **Tasigna** | Cyprus | 1st line | No | - |
| **Tasigna** | Estonia | 1st line | No | - |
| **Tasigna** | Kosovo | 1st line | No | - |
| **Tasigna** | Latvia | 1st line | No | - |
| **Tasigna** | Macedonia | 1st line | No | - |
| **Tasigna** | Poland | 1st line | No | - |
| **Tasigna** | Serbia | 1st line | No | - |
| **Tasigna** | Slovakia | 1st line | Yes | Jan-12 |
| **Tasigna** | Slovenia | 1st line | Yes | Nov-11 |
| **Tasigna** | Sweden | 1st line | 150 mg is reimbursed in 1st line only (200 mg is NOT reimbursed 1st line) | Oct-11 |
| **Tasigna** | Switzerland | 1st line | Yes | Oct-10 |
| **Tasigna** | United Kingdom | 1st line | Yes | Apr-12 |
| **Tasigna** | Belgium | 1st line | Yes | Jul-11 |
| **Tasigna** | Finland | 1st line | Yes | Sep-11 |
| **Tasigna** | Germany | 1st line | Yes | Jan-11 |
| **Tasigna** | Greece | 1st line | Yes | May-12 |
| **Tasigna** | Hungary | 1st line | Yes | Feb-14 |
| **Tasigna** | Ireland | 1st line | Yes | Jun-11 |
| **Tasigna** | Israel | 1st line | Yes | Apr-11 |
| **Tasigna** | Luxembourg | 1st line | Yes | - |
| **Tasigna** | Netherlands | 1st line | Yes | Jul-05 |
| **Tasigna** | Spain | 1st line | Yes | Jul-11 |
| **Tasigna** | France | 1st line | Yes | Feb-13 |
| **Tasigna** | Norway | 1st line | Yes | Jan-14 |
| **Tasigna** | Portugal | 1st line | Yes | Aug-13 |
| **Tasigna** | Bulgaria | 1st line | Yes | Mar-12 |
| **Tasigna** | Iceland | 1st line | Yes | Mar-13 |
| **Tasigna** | Romania | 1st line | Yes | Jul-14 |
| **Tasigna** | Austria | 1st line | No | - |
| **Tasigna** | Italy | 1st line | Yes | Nov-11 |
| **Tasigna** | Denmark | 1st line | Yes | EMA |
| **Tasigna** | Lithuania | 1st line | Yes | Jun-15 |
| **Tasigna** | Montenegro | 1st line | No | - |
| **Tasigna** | Czech Republic | 1st line | Yes | Jul-12 |
| **Tasigna** | Bosnia | 1st line | Yes | Mar-12 |
| **Tasigna** | Malta | 1st line | Yes | Feb-13 |
| **Tasigna** | Kosovo | 2nd line | No | - |
| **Tasigna** | Macedonia | 2nd line | No | - |
| **Tasigna** | Slovakia | 2nd line | Yes | Jan-12 |
| **Tasigna** | Slovenia | 2nd line | Yes | May-09 |
| **Tasigna** | Spain | 2nd line | Yes | Jun-08 |
| **Tasigna** | Sweden | 2nd line | Both 150mg and 200mg is reimbursed 2nd line | Oct-11 |
| **Tasigna** | Switzerland | 2nd line | Yes | Aug-07 |
| **Tasigna** | United Kingdom | 2nd line | Yes | Jan-12 |
| **Tasigna** | Belgium | 2nd line | Yes | Sep-08 |
| **Tasigna** | Estonia | 2nd line | Yes | Oct-09 |
| **Tasigna** | Finland | 2nd line | Yes | Sep-08 |
| **Tasigna** | Germany | 2nd line | Yes | Jan-08 |
| **Tasigna** | Greece | 2nd line | Yes | May-08 |
| **Tasigna** | Hungary | 2nd line | Yes | Aug-08 |
| **Tasigna** | Ireland | 2nd line | Yes | Jun-08 |
| **Tasigna** | Israel | 2nd line | Yes | Feb-13 |
| **Tasigna** | Lithuania | 2nd line | Yes | Oct-12 |
| **Tasigna** | Luxembourg | 2nd line | Yes | - |
| **Tasigna** | Netherlands | 2nd line | Yes | Jun-05 |
| **Tasigna** | France | 2nd line | Yes | Apr-08 |
| **Tasigna** | Croatia | 2nd line | Yes | Jun-10 |
| **Tasigna** | Norway | 2nd line | Yes | Nov-11 |
| **Tasigna** | Portugal | 2nd line | Yes | Nov-10 |
| **Tasigna** | Poland | 2nd line | Yes | Jan-10 |
| **Tasigna** | Bulgaria | 2nd line | Yes | Mar-12 |
| **Tasigna** | Iceland | 2nd line | Yes | Jun-08 |
| **Tasigna** | Romania | 2nd line | Yes | Jul-14 |
| **Tasigna** | Austria | 2nd line | No | - |
| **Tasigna** | Malta | 2nd line | Yes | Oct-11 |
| **Tasigna** | Latvia | 2nd line | Yes | Feb-11 |
| **Tasigna** | Italy | 2nd line | Yes | Aug-08 |
| **Tasigna** | Denmark | 2nd line | Yes | EMA |
| **Tasigna** | Albania | 2nd line | Yes | Mar-13 |
| **Tasigna** | Serbia | 2nd line | Yes | Jul-11 |
| **Tasigna** | Montenegro | 2nd line | No | - |
| **Tasigna** | Czech Republic | 2nd line | Yes | Jul-08 |
| **Tasigna** | Bosnia | 2nd line | Yes | Mar-12 |
| **Tasigna** | Cyprus | 2nd line | Yes | Jun-09 |

***SUPPLEMENTARY MATERIAL***

***EUROCARE-6 patient selection***

In accordance with the EUROCARE-6^22^ and HAEMACARE^61^ morphology groupings, we first selected all patients diagnosed with hematological malignancies in 2000-2013 aged >15 and ≤64 (555,142 cases) using disease codes based on the third edition of the International Classification of Disease for Oncology (ICD-O-3) (Table 1 - Suppl Mat), and then patients with myeloid malignancies (113,322 cases): mastocytosis (codes 9740, 9741, and 9742); CML (codes 9863 [CML with no information about cytogenetics or CML NOS] and 9875 [Abelson (Abl) oncogene with breakpoint cluster region (Bcr) translocation, *BCR-ABL1* positive CML]); myeloproliferative neoplasms (MPNs, codes 9950 and 9960-9964, 9966); acute myeloid leukemia (AML, codes 9840, 9861, 9865, 9867, 9869-9874, 9891, 9895-9898, 9910-9911, 9920, and 9930-9931, 9984, 9987); acute promyelocytic leukemia (APL, code 9866); myelodysplastic syndrome (MDS, codes 9980, 9982-9983, 9985-9986, 9989, 9991-9992); myelodysplastic/myeloproliferative syndrome (MDS/MPN, codes 9876, 9945-9946 [including cases of Ph-negative atypical CML (code 9876) which are not targeted by TKIs], and 9975); myeloid not otherwise specified (NOS, codes 9800, 9801, 9805-9809, and 9860)^60^. No CR exclusion based on the proportion (≤14%) of cases recorded as death certificate only (DCO) or autopsy cases was made. We excluded CRs with partial coverage in 2000-2006 (only two years of incidence data in the reference period) and CRs with high proportion of cases lost to follow-up (>10%). Subsequently, only the CRs with ≥70% of myeloid cases with known microscopic verification and ≤30% of myeloid NOS cases (ICD-O-3 codes 9800, 9801, 9805 and 9860) were included in the analyses. Overall, 18,083 CML cases were analysed.
