# Supplementary material for: Clear Improvement in Real-World Chronic Myeloid Leukemia Survival: A Comparison With Randomized Controlled Trials
Source: Front Oncol. 2022 Jul 14;12:892684. doi: 10.3389/fonc.2022.892684 (PMC9333088; doi:10.3389/fonc.2022.892684)
Supplement: Supplementary file 2 [file DataSheet_2.pdf]

Calendar Year	Europe			European Country																						
	Eastern Countries[1]	Non-Eastern Countries[2]	All Countries[3]	Austria	Belgium	Bulgaria	Switzerland	Czech Republic	Germany	Denmark	Estonia	Spain	Finland	France	UK-Northern Ireland	Croatia	Ireland	Italy	Lithuania	Latvia	Netherlands	Norway	Poland	Portugal	Slovenia	Slovakia
**2000**	91	970	1061	25	33	0	31	22	300	13	1	90	17	115	129	0	4	150	1	0	51	12	64	0	0	3
**2001**	86	680	766	19	9	0	13	28	228	9	0	79	18	52	90	0	3	116	0	0	34	9	53	0	1	5
**2002**	85	666	751	17	23	0	12	21	237	11	0	57	12	65	120	1	2	63	0	0	30	3	55	9	4	9
**2003**	103	491	594	11	14	1	14	31	142	3	2	33	10	61	101	2	4	55	1	0	21	10	62	7	3	6
**2004**	82	452	534	12	15	0	11	21	113	6	0	32	16	71	72	2	3	58	2	0	28	7	55	1	5	4
**2005**	84	353	437	10	11	0	8	25	97	7	1	20	3	48	77	0	2	45	3	0	21	0	53	3	1	2
**2006**	58	326	384	6	12	0	11	14	84	4	1	16	8	49	71	1	1	39	5	0	14	4	37	5	1	1
**2007**	44	248	292	5	5	0	4	12	63	9	1	18	8	32	50	2	2	29	9	0	13	1	21	7	0	1
**2008**	35	242	277	5	7	0	8	12	63	3	2	15	2	35	41	2	0	40	2	1	13	2	17	6	0	1
**2009**	17	261	278	6	14	0	3	3	61	5	0	17	2	41	44	1	2	42	2	0	18	3	10	2	0	2
**2010**	31	264	295	4	8	0	5	11	63	4	1	15	2	50	42	2	3	39	1	0	18	4	17	5	0	1
**2011**	20	268	288	10	6	0	5	4	64	1	0	15	6	33	57	3	4	34	4	1	22	4	9	3	1	2
**2012**	25	231	256	4	10	2	8	8	59	4	2	12	6	32	32	1	2	36	2	0	14	3	10	8	0	1
**2013**	20	235	255	3	7	0	3	6	50	5	0	26	3	32	50	2	3	35	1	0	11	3	13	2	0	0
**2014**	39	229	268	4	10	0	6	11	50	6	0	14	4	32	45	3	1	36	5	0	14	2	22	2	0	1
**2015**	29	228	257	7	6	0	6	10	46	1	0	15	5	45	46	7	4	28	2	0	9	1	15	1	1	2
**2016**	27	212	239	2	5	0	11	3	40	3	0	19	3	34	49	0	2	21	6	0	14	3	17	3	3	1
**2017**	18	180	198	4	5	0	3	5	49	4	0	15	3	16	33	1	1	29	1	0	11	2	12	4	0	0
**2018**	16	199	215	5	7	0	4	4	53	5	1	17	1	34	36	0	2	23	2	0	9	0	9	2	1	0
**2019**	25	214	239	6	7	0	10	2	47	4	1	20	4	23	55	1	0	21	2	0	10	3	19	1	2	1
**2020**	12	200	212	9	7	0	7	5	51	4	1	12	1	21	37	3	1	22	1	0	18	5	5	1	1	0
**2021**	13	173	186	4	5	0	4	6	39	2	0	12	1	15	45	0	2	31	0	0	11	2	7	0	0	0
**2022**	0	7	7	1	0	0	0	0	1	0	0	0	0	1	0	0	0	4	0	0	0	0	0	0	0	0
**Total**	**960**	**7329**	**8289**	**179**	**226**	**3**	**187**	**264**	**2000**	**113**	**14**	**569**	**135**	**937**	**1322**	**34**	**48**	**996**	**52**	**2**	**404**	**83**	**582**	**72**	**24**	**43**

Suppl Mat Table 6. Allogeneic bone marrow transplantations for CML in European countries covered by the study by calendar year and country (data provided by prof. Yves Chalandon and Mrs Laurien Baaij for the EBMT CMWP as personal communication).

Chronic Malignancies Working Party (CMWP); CML: chronic myeloid leukemia; EBMT, European Society for Blood and Marrow Transplantation (EBMT).

[1] Eastern European countries (Bulgaria, Czech Republic, Estonia, Latvia, Lithuania, Poland, Slovakia).

[2] Non-Eastern European countries (Austria, Belgium, Switzerland, Germany, Denmark, Spain, Finland, France, UK and Northern Ireland, Croatia, Ireland, Italy, Netherlands, Norway, Portugal, Slovenia).

[3] All European countries (Eastern European countries[1] plus Non-Eastern European countries[2]).
